# Cost-effectiveness analysis of malaria rapid diagnostic test incentive schemes for informal private healthcare providers in Myanmar

**DOI:** 10.1186/s12936-015-0569-7

**Published:** 2015-02-05

**Authors:** Ingrid T Chen, Tin Aung, Hnin Nwe Nwe Thant, May Sudhinaraset, James G Kahn

**Affiliations:** Global Health Sciences, University of California, San Francisco, 550 16th Street, 3rd Floor, San Francisco, CA 94158 USA; Population Services International Myanmar, No 16, Shwe Gon Taing Street 4, Yangon, Myanmar

**Keywords:** Cost-effectiveness, Malaria, Rapid diagnostic test, Artemisinin combination therapy, Subsidy, Behaviour communication change, Drug resistance, Informal provider, Myanmar

## Abstract

**Background:**

The emergence of artemisinin-resistant *Plasmodium falciparum* parasites in Southeast Asia threatens global malaria control efforts. One strategy to counter this problem is a subsidy of malaria rapid diagnostic tests (RDTs) and artemisinin-based combination therapy (ACT) within the informal private sector, where the majority of malaria care in Myanmar is provided. A study in Myanmar evaluated the effectiveness of financial incentives *vs* information, education and counselling (IEC) in driving the proper use of subsidized malaria RDTs among informal private providers. This cost-effectiveness analysis compares intervention options.

**Methods:**

A decision tree was constructed in a spreadsheet to estimate the incremental cost-effectiveness ratios (ICERs) among four strategies: no intervention, simple subsidy, subsidy with financial incentives, and subsidy with IEC. Model inputs included programmatic costs (in dollars), malaria epidemiology and observed study outcomes. Data sources included expenditure records, study data and scientific literature. Model outcomes included the proportion of properly and improperly treated individuals with and without *P. falciparum* malaria, and associated disability-adjusted life years (DALYs). Results are reported as ICERs in US dollars per DALY averted. One-way sensitivity analysis assessed how outcomes depend on uncertainty in inputs.

**Results:**

ICERs from the least to most expensive intervention are: $1,169/DALY averted for simple subsidy *vs* no intervention, $185/DALY averted for subsidy with financial incentives *vs* simple subsidy, and $200/DALY averted for a subsidy with IEC *vs* subsidy with financial incentives. Due to decreasing ICERs, each strategy was also compared to no intervention. The subsidy with IEC was the most favourable, costing $639/DALY averted compared with no intervention. One-way sensitivity analysis shows that ICERs are most affected by programme costs, RDT uptake, treatment-seeking behaviour, and the prevalence and virulence of non-malarial fevers. In conclusion, private provider subsidies with IEC or a combination of IEC and financial incentives may be a good investment for malaria control.

**Electronic supplementary material:**

The online version of this article (doi:10.1186/s12936-015-0569-7) contains supplementary material, which is available to authorized users.

## Background

Artemisinin resistance threatens worldwide efforts to control and eliminate malaria. Artemisinin is the most effective first-line anti-malarial against *Plasmodium falciparum* parasites, and the first signs of drug resistance were seen in Cambodia since 2006 [1,2]. By 2012, artemisinin resistance was seen in the Thai-Myanmar border area [3]. This finding is a call for action, as it is reminiscent of the worldwide spread of chloroquine resistance [4,5]. In order to ensure that the front-line anti-malarial remains efficacious for the 216 million individuals infected with malaria throughout the world each year, aggressive efforts to curb artemisinin resistance are critical [6].

An estimated 37.4 million people in Myanmar live in malaria-endemic areas, where 74% of malaria cases are *P. falciparum* infections and 26% are *Plasmodium vivax* infections [7]. Malaria is prevalent year-round and the risk of infection is mostly in rural, forested areas at altitudes of less than 1,000 m [7,8]. There is a wide range of uncertainty in the incidence of malaria in Myanmar, as the large number of cases from migrants working in forests and agricultural areas are difficult to quantify. Recent estimates of the number of cases range between 500,000 cases (in 2011) to 4.2 million cases (in 2008), resulting in 9,100 deaths annually [9-11].

In Myanmar, a combination of unregulated drugs and their overuse due to symptomatic diagnosis are major sources of pressure on drug resistance [7,12]. Drug resistance may be selected when parasites survive exposure to low levels of drugs [13], which in Myanmar is encouraged by counterfeit drugs that contain sub-therapeutic doses of artemisinin monotherapy [14-17], and providers that prescribe artemisinin monotherapy, especially at lower doses than recommended to cure malaria [18]. Both of these practices are common in the largely unregulated private sector, where 40-80% of the population seeks health care [7,12].

Within the Myanmar informal private sector, common practices of itinerant drug vendors and pharmacists include the overuse of artemisinin-family compounds through symptomatic diagnosis and their misuse through the sale of artemisinin monotherapy [7]. These practices are a result of the prohibitively high costs of malaria rapid diagnostic tests (RDTs) and artemisinin-based combination therapy (ACT), which includes a long-acting anti-malarial to lessen the selective pressure for artemisinin drug resistance. The targeting of donor funds for RDTs and ACT in the private sector therefore offers the potential to drive the proper use of artemisinin-based drugs in Myanmar.

The subsidy of ACT and RDTs for use in the private sector is expected to be the most important intervention to prevent the spread of drug resistance in Myanmar [18]. As RDTs do not require specialized training or equipment, the provision of subsidized RDTs with a training session can improve diagnostic accuracy [19]. Also, subsidized ACT is quality controlled and if sold at lower prices than other anti-malarials, can replace artemisinin monotherapy.

Although ACT subsidies in Africa and Asia are increasing in number, to date only Cambodia has deployed both RDTs and ACT to the private sector at a national scale [20]. The Cambodia programme, which started in 2002, showed that RDT uptake was slow, reaching the highest levels seven years after the intervention was introduced, although there was no data on the quality of RDTs or provider adherence to test results [20]. The Cambodian experience suggested that future RDT subsidies to informal providers should include a training programme emphasizing the importance of RDTs and their proper use, and that financial incentives could also be explored to promote RDT uptake [20]. A recent pilot study was conducted in rural Myanmar that builds on these suggestions, in addition to incorporating community education to drive consumer demand for RDTs. The pilot study also explored the use of information, education and counselling (IEC) sessions to encourage RDT use among informal private providers.

The exploration of financial incentives *vs* IEC for subsidized malaria RDT use in the informal private sector is novel, and the World Health Organization (WHO) recommends that the cost-effectiveness of new interventions be established before strategies are recommended as policy [18]. The objective of this study is to assess the cost-effectiveness of three subsidy strategies: a simple subsidy, subsidy with financial incentives and subsidy with IEC, to drive the appropriate use of malaria RDTs among informal private providers in Myanmar.

## Methods

### Model overview

**Figure 1:**
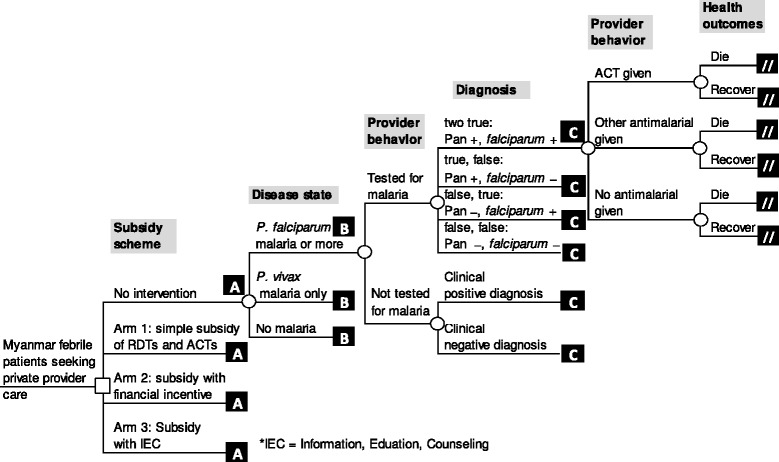
**Decision tree model for malaria rapid diagnostic test subsidy schemes.**

The cost-effectiveness for a RDT subsidy scheme pilot study was estimated using a decision tree model constructed in a spreadsheet (Excel, Microsoft 2010). The decision tree compared three intervention arms with ‘no intervention’ (Figure 1), considering programme and medical costs from a societal perspective. The decision tree comprises of a decision node among the subsidy approaches, followed by chance nodes based on the true disease status, followed by test and treatment consequences. The model estimates the health and economic value of each outcome (detailed in Additional file 1).

Costs included programmatic costs (overhead, staff, travel, and supply costs) and patient commodity costs, and were based on Population Services International (PSI) Myanmar’s finance and account records. Cost records comprised of PSI overhead costs scaled to the pilot study, actual pilot study costs, staffing costs from the artemisinin monotherapy replacement (AMTR) programme (scaled to the number of outlets reached by the pilot study), as well as commodity costs from other programmes (detailed in Additional file 1). The exchange rate used was 907 Kyat/USD, from 1 May, 2013.

Effectiveness was estimated in disability-adjusted life years (DALYs), based on the health effects of malaria and non-malarial fevers and appropriateness of the medicine prescribed. DALYs resulting from death were calculated based on the years lost from mortality, based on the difference between mean life expectancy and average age of malaria deaths, with a 3% discount rate applied to each year in the future. DALYs for surviving patients were calculated by multiplying the average duration of illness with the disability weight for the illness, based on published disability levels [21,22] (detailed in Additional file 1). For every path of events in the decision tree, the associated health outcomes were weighted by probability, and the total DALYs incurred was a sum of the weighted health outcomes.

Cost-effectiveness was measured incrementally, as the cost per DALY averted for the study population, the number of patients that seek private provider care at all outlets enrolled in the RDT pilot study from May to September, 2013. ICERs showed that adding programmes led to increased costs that were compensated by large increases in effectiveness. Due to this scenario, which is called ‘extended dominance’, the cost-effectiveness of each option was also compared with no intervention (further detail on extended dominance available in Additional file 1). Model assumptions are described in Additional file 1, and do not account for onward malaria transmission (from untreated cases) or the possibility of selection for drug resistance.

### Programme description

The pilot study took place in the Mon and Shan states in rural Myanmar. The study recruited approximately 600 private providers between April and September 2013. Three types of private providers were recruited: providers in general retail stores, itinerant drug vendors and medical drug representatives. All providers had been receiving subsidized ACT since September 2012 through PSI Myanmar’s AMTR programme, which aims to crowd out artemisinin monotherapy with subsidized quality-assured ACT.

Townships were selected on the basis of similar population size, malaria burden and socio-economic characteristics, and intervention arms were randomized at a township level. The three pilot study arms were:

- Arm 1: simple subsidy of RDTs for sale at 100 Kyat (approximately $0.12) to retailers with a support visit every month
- Arm 2: RDT subsidy with financial incentive: one free RDT for every five purchased with a support visit every month
- Arm 3: RDT subsidy with support visits every two weeks.

Prior to RDT rollout, community sensitization activities were also performed in all pilot study locations.

### Data input values

For data inputs (Tables 1, 2 and 3), each study arm was normalized to target 600 outlets. Data inputs employed a combination of finance/account records and management information systems (MIS) data from PSI Myanmar, quantitative and qualitative data from the pilot study, and a review of published scientific literature (detailed in Additional file 1). RDT uptake and the treatment prescribed were measured through the RDT pilot study, which used four analytical methods: 1) household surveys, 2) interviews with private providers, 3) mystery client visits, and 4) stock audit data from supply points. The household surveys measured RDT uptake at a community/patient level before and after the intervention. The interviews with private providers included a survey of anti-malarial and RDT stock and prices. RDT uptake was also measured through mystery clients, who presented with an alleged fever to enrolled providers in the beginning and end of the pilot study. Stock audit data monitored RDT and ACT use, including RDT results which the programme required be written on the RDT, and returned to supply points for resupply. The literature search comprised of a series of Web of Knowledge and Google Scholar searches conducted between November 2012 and April 2013. Keywords included malaria, Myanmar, drug adherence, artemisinin, and subsidy; all references were screened for potential relevance to this study.

**Table 1:** **Base case data inputs for epidemiology, health outcomes and diagnostic test characteristics**

**Parameter**		**Input value**	**Source**
**Epidemiology**			
Percentage of *P. falciparum (P.f.)*/*P. vivax (P.v.)* malaria		65% *P.f./*35% *P.v.*	Published data [23], PSI Myanmar stock audit data
Proportion of febrile cases in population that are malaria		8%	PSI Myanmar MIS data (Sun Primary Health)
Average number of febrile patients that visit one private provider per month		20	PSI Myanmar MIS data (Sun Primary Health)
**Health outcomes**			
Case fatality rates for *P. falciparum* malaria	Given ACT	0.01%	Very low probability
	Given chloroquine or quinine	0.7%	Published data on falciparum drug resistance [24]
	Given no anti-malarial	3%	Published case fatality rates in Bago [25] and on eastern border of Myanmar [26]
Case fatality rates for *P. vivax*	Given ACT	0.01%	Very low probability
	Given chloroquine or quinine	0.01%	Published *P. vivax* treatment rates with chloroquine in Papua [27]
	Given no anti-malarial	1%	Extrapolated from published materials from Papua [28]
Case fatality rate for non-malarial febrile illnesses	Given ACT or other anti-malarial	0.2%	Published data from Bago, Myanmar [25] triangulated with PSI MIS data
	Given no anti-malarial	0.16%	Published data on burden of disease in Myanmar [29] and non-malarial fevers in Laos [30]
Average duration of malaria illness without effective treatment		1 week	Published data on hospital records in Myanmar [31]
Average duration of non-malarial febrile illness		1 week	Assumption
DALY weight of malaria		0.2	Published data [21]
DALY weight of non-malarial fever		0.18	Estimated from published data [22]
Mean life expectancy in Myanmar		62 years	Average from 3 studies [32-34]
Average age of malaria-induced death in intervention townships		25 years	PSI Myanmar MIS data
Average age of non-malarial febrile death in Myanmar		30 years	PSI Myanmar MIS data
Discount rate		3%	Standard rate
**Diagnostic test characteristics**			
RDT sensitivity and specificity	*P. falciparum* sensitivity	100%	Published RDT performance [35]
	*P. falciparum* specificity	97%	Published RDT performance [35]
	Pan *plasmodium* sensitivity	92%	Published RDT performance [35]
	Pan *plasmodium* specificity	98%	Published RDT performance [35]

**Table 2:** **Base case inputs for provider behaviour** *

**Provider behaviour**		**No intervention**	**Arm 1**	**Arm 2**	**Arm 3**
Probability of clinical diagnosis		0.98	0.98	0.98	0.92
Probability of using RDT		0.02	0.02	0.02	0.08
Diagnosis	Medicine prescribed				
Clinical Diagnosis	ACT	0.05	0.12	0.12	0.19
	Other anti-malarial	0.03	0.07	0.07	0.07
	No anti-malarial	0.92	0.81	0.81	0.74
RDT Pan + *falciparum* +	ACT	0.75	0.78	0.84	0.87
	Other anti-malarial	0.05	0.05	0.05	0.05
	No anti-malarial	0.2	0.17	0.11	0.08
RDT Pan + *falciparum* -	ACT	0.5	0.10	0.10	0.10
	Other anti-malarial	0.25	0.45	0.45	0.45
	No anti-malarial	0.25	0.45	0.45	0.45
RDT Pan - *falciparum* +	ACT	0.75	0.78	0.84	0.87
	Other anti-malarial	0.05	0.05	0.05	0.05
	No anti-malarial	0.2	0.17	0.11	0.08
RDT Pan - *falciparum* -	ACT	0.4	0.057	0.083	0.022
	Other anti-malarial	0.02	0.029	0.056	0.089
	No anti-malarial	0.58	0.914	0.861	0.889

*Source: pilot study data from household survey, mystery client visits, provider demographics from in-depth qualitative interviews, and PSI Myanmar MIS data.

‘No antimalarial’ comprises of the use of antipyretics 70% of the time and antibiotics 30% of the time, as described and rationalized in the Additional File section ‘Assumptions’.

Note: baseline RDT uptake is a conservative lower bound based on household surveys with denominator adjusted to only include care sought from informal providers. Mystery clients were prompted to suggest they have malaria, possibly motivating providers to use RDTs at higher rates than in real-life scenarios.

**Table 3:** **Costs for rapid diagnostic test intervention**

**Annual direct programme first year costs (non-recurrent in italics)**				
**Costs for RDT intervention, 600 providers**	**No intervention**	**Arm 1**	**Arm 2**	**Arm 3**
*Interpersonal communicators*	$0	*$34,599*	*$34,599*	*$34,599*
Jr Health Service Officers	$0	$17,568	$17,568	$17,568
Product promoters	$0	$31,374	$31,374	$62,748
Office personnel	$0	$79,186	$79,186	$79,186
*Incentives for providers*	*$0*	*$17,784*	*$17,784*	*$17,784*
Commodities	$95,614	$103,658	$104,087	$119,127
Materials for providers	$0	$19,656	$19,656	$19,656
Materials for product promoters	$0	$324	$324	$324
*Field staff training*		*$6,951*	*$6,951*	*$6,951*
Field staff transport: monthly office visits	$0	$30,834	$30,834	$53,028
Motorcycle taxi	$0	$39,202	$39,202	$59,402
PSI Overhead	$0	$5,329	$5,329	$5,329
Shipping logistics	$0	$1,271	$1,271	$1,271
**Total, year 1**	**$95,614**	**$387,735**	**$388,163**	**$476,973**
**Non-recurrent, year 1**	**$0**	**$59,334**	**$59,334**	**$59,334**
**Recurrent annual**	**$95,614**	**$328,401**	**$328,829**	**$417,639**
**Commodity cost per unit**				
	**No intervention**	**Arm 1**	**Arm 2**	**Arm 3**
RDT societal cost (donor + patient)	$1.16	$0.68	$0.80	$0.68
ACT	$1.65	$1.65	$1.65	$1.65
Quinine and chloroquine	$0.55	$0.55	$0.55	$0.55
‘No anti-malarial’ (70% antipyretics, 30% antibiotics)	$0.58	$0.58	$0.58	$0.58
**Patient and provider time and travel costs**				
**Costs for RDT intervention, 600 providers**	**No intervention**	**Arm 1**	**Arm 2**	**Arm 3**
Patient and provider time costs	$0	$53,366	$53,366	$64,498
Provider travel costs to restock RDTs	$0	$79,344	$79,344	$79,344

### Sensitivity analysis

One-way sensitivity analysis was performed on each input value, and results were ranked as inputs that most affect costs, and inputs that most affect health outcomes. Base case results represent a lower bound estimate for RDT uptake, and the rationale for the sensitivity analysis ranges explored are detailed in Additional file 1. The ICERs were calculated and ranked, and significant inputs, defined as affecting an absolute change in cost by more than $14,400 ($100 per 1,000 individuals annually), and/or an absolute change in more than 3,600 DALYs (25 DALYs per 1,000 individuals annually) across the study population, were reported.

### Ethical considerations

In 2012, the Myanmar Ministry of Health ceded authority to the AMTR project, which includes the RDT pilot study, to PSI Myanmar. The RDT pilot study received approval from the PSI Research Ethics Board (REB). The members of the study team from the University of California, San Francisco did not interact with patients.

## Results

### Costs

**Figure 2:**
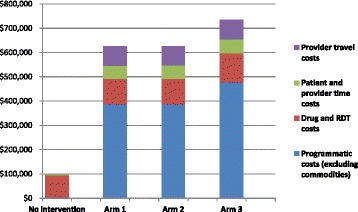
**Categorized societal costs (annual).**

The costs are divided into commodities, programme expenses (mainly staff), time, and travel (Table 4 shows year 1, subsequent years detailed in Additional file 1). Programmatic expenses were the main expense, comprising of over half of the costs for each study arm (Figure 2). For the total study population (144,000 individuals annually; 600 providers * 20 febrile patients per provider monthly * 12 months/year), a simple subsidy was also the least expensive ($625,486, $4,300 per 1,000 individuals annually), with the addition of financial incentives requiring minor increases in price ($626,342, $4,350 per 1,000 individuals annually), and the incorporation of IEC requiring further expenses ($734,339, $5,100 per 1,000 individuals annually).

**Table 4:** **Annual commodities, programmatic expenses, time and travel cost**

**Scenario (societal)**	**Total cost**	**Drug and RDT costs (scaled to uptake)**		**Programmatic staff and non-commodity costs**	**Patient and provider time costs** *	**Provider travel costs** **
		**Total**	**(RDT donor only)**			
**No intervention**	$96,996	$95,614	**$0**	**$0**	$1,382	$0
**Arm 1: simple subsidy**	$625,486	$103,658	**$1,037**	**$387,735**	$54,748	$79,344
**Arm 2: subsidy with financial incentive**	$626,342	$104,087	**$1,382**	**$388,163**	$54,748	$79,344
**Arm 3: subsidy with IEC**	$734,339	$119,127	**$4,147**	**$476,973**	$58,896	$79,344

*Includes time spent conducting RDT, and provider time for monthly supply point visit based on wages, as providers were not compensated by the programme.

**Patient travel costs were excluded and were the same across each arm, estimated to be $504,000 per arm.

Bold = donor costs.

The RDT donor costs reflect a $0.36 subsidy per unit from the donor for arms 1 and 3, and a $0.48 subsidy per unit for arm 2. These are scaled to a base case uptake of 2% for arms 1 and 2, and 8% for arm 3 (Table 2). Total commodity costs include all patient purchases: $0.32 per RDT, $0.53 per subsidized ACT (donor costs not included in analysis), $0.55 per course of ‘other anti-malarial’ (quinine or chloroquine), and $0.58 per course of ‘no anti-malarial’ (weighted as 70% antipyretic/30% antibiotic). The donor RDT cost is also specified for programmatic planning.

### Health outcomes

Health outcomes are reported as DALYs (Table 5). A simple subsidy offered reasonable improvements compared to no intervention, amounting to 452 DALYs averted (3.14 DALYs averted per 1,000 individuals annually). The financial incentive led to a very minor improvement of only 5 DALYs averted compared to a simple subsidy (0.035 DALYs averted per 1,000 individuals annually). The most substantial improvement was provided by the IEC strategy, providing 540 DALYs averted (536 due to deaths averted, four due to morbidity averted) compared to the subsidy with financial incentive. This translates to 3.75 DALYs averted per 1,000 individuals annually. A simple RDT subsidy, therefore, improved health outcomes, with effects further enhanced through an IEC strategy.

**Table 5:** **Cost-effectiveness ratios from a societal perspective for first year of intervention**

**Subsidy scheme**	**Total costs**	**Added costs** ***vs*** **prior strategy**	**Total DALYs incurred**	**DALYs averted** ***vs*** **prior strategy**	**Incremental cost per DALY averted** ***vs*** **prior strategy**	**Cost per DALY averted** ***vs*** **no intervention**
**No intervention**	$96,996	--	10,155	--	--	--
**Arm 1: Simple subsidy**	$625,486	$528,490	9,703	452	$1,169	$1,169
**Arm 2: Subsidy with financial incentive**	$626,342	$857	9,698	5	$185	$1,159
**Arm 3: Subsidy with IEC**	$734,339	$107,997	9,158	540	$200	$639

### Incremental cost-effectiveness ratios

The base case ICERs for year one are: $1,169/DALY averted for the simple RDT subsidy compared to no intervention, $185/DALY averted for RDT subsidy with financial incentives compared to simple subsidy, and $200/DALY averted for subsidy with IEC compared to subsidy with financial incentive (Table 5 shows year 1, subsequent years are shown in Additional file 1).

**Figure 3:**
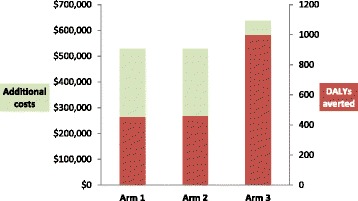
**Cost and DALYs averted** ***vs*** **no intervention.**

Since the ICER decreases from the first comparison (simple subsidy *vs* no intervention, $1,169/DALY) to the second comparison (subsidy with incentives *vs* simple subsidy, $185/DALY), representing extended dominance, study arms were also compared directly with no intervention (Table 5). The result was $1,169/DALY averted for a simple subsidy, $1,159/DALY averted for a subsidy with financial incentives, and $639/DALY averted for a subsidy with IEC. The costs and health benefits of the simple subsidy proved to be very similar to the subsidy with financial incentives, while IEC showed increases in cost offset by large improvements in health outcomes (Figure 3).

### Sensitivity analysis

One-way sensitivity analysis shows that the analysis is driven by the levels of RDT uptake, levels of treatment-seeking within outlets, programmatic costs, drug costs, the prevalence and virulence of non-malarial fevers, and the virulence of *P. falciparum* malaria (Table 6, described below and detailed in Additional file 1).

**Table 6:** **One-way sensitivity analysis summary for year 1 costs** *

**Parameter**	**Value (Low, high of range)**	Order**	**ICERs ($/DALY averted)**		
			**Low, high of range**		
			**2** ^**nd**^ ***vs*** **1** ^**st**^	**3** ^**rd**^ ***vs*** **2** ^**nd**^	**4** ^**th**^ ***vs*** **3** ^**rd**^
**Inputs that affect costs and health outcomes**					
Base case	N/A	C, 1, 2, 3	$1,169	$185	$200
*Probability of using RDT in arm 1 (base case 0.02)*	*0.00*	*C, 1, 2, 3*	*$1,389*	*$53*	*$200*
	*0.65*	*C, 2, 1, 3*	*1,159*	*$45*	*Dominated (DOM)*
*Probability of using RDT in arm 2 (base case 0.02)*	*0.00*	*C, 2, 1, 3*	*$1,390*	*$39*	*$200*
	*0.65*	*C, 1, 3, 2*	*$1,169*	*$3*	*$2*
Probability of using RDT in arm 3 (base case 0.08)	0.02	C, 1, 2, 3	$1,169	$185	$317
	0.65	C, 1, 2, 3	$1,169	$185	$72
Number of febrile patients seeking care per private sector provider per month (base case 40)	1	C, 1, 2, 3	$23,046	$1,939	$3,323
	40	C, 1, 2, 3	$594	$139	$118
Probability of ‘no anti-malarial’ administration for clinical diagnosis, arm 1 (base case 0.81)**	*0.5*	*C, 2, 1, 3*	*$1,159*	*$34*	*DOM*
	*0.93*	*C, 1, 2, 3*	*DOM*	*$1,159*	*$200*
Probability of ‘no anti-malarial’ administration for clinical diagnosis, arm 2 (base case 0.81)***	*0.5*	*C, 1, 2, 3*	*$1,169*	*$35*	*DOM*
	*0.93*	*C, 2, 1, 3*	*DOM*	*$1,169*	*$200*
Probability of ‘no anti-malarial’ administration for clinical diagnosis, arm 3 (base case 0.74)***	0.5	C, 1, 2, 3	$1,169	$185	$93
	0.93	C, 1, 2, 3	$1,169	$185	DOM
Probability of ACT administration for clinical diagnosis, no intervention (base case 0.05)***	0.05	C, 1, 2, 3	$1,169	$185	$200
	0.4	C, 1, 2, 3	DOM	$200	$200
**Inputs that affect costs only**					
Programme costs per febrile individual (base case $0 for C, $3.61 for arms 1 and 2, and $4.23 for arm 3)	$2	C, 1, 2, 3	$655	$92	$36
	$10	C, 1, 2, 3	$3,204	$92	$36
Cost of ‘no anti-malarial’ (base case $0.58)	$0.30	C, 1, 2, 3	$1,178	$194	$205
	$1.00	C, 1, 2, 3	$1,156	$272	$193
Cost of ACT, same across all arms (base case $1.65)	$0.50	C, 2, 1, 3	$1,148	$179	$99
	$2.50	C, 1, 2, 3	$1,186	$199	$214
Probability of using RDT for no intervention (base case 0.02)	0.02	C, 1, 2, 3	$1,169	$185	$200
	0.11	C, 1, 2, 3	$4,183	$185	$200
Cost of other anti-malarial, same across all arms (base case $0.55)	$0.18	C, 1, 2, 3	$1,165	$179	$200
	$1.65	C, 1, 2, 3	$1,183	$201	$201
**Inputs that affect health outcomes only**					
Probability of death for non-malarial fever given no anti-malarial (base case 0.0016)	0.001	C, 1, 2, 3	$1,873	$297	$247
	0.05	C, 1, 2, 3	$37	$6	$12
Probability of death for non-malarial fever given ACT (base case 0.002)	0.001	C, 1, 2, 3	$846	$140	$153
	0.05	C, 1, 2, 3	DOM	DOM	DOM
Percentage of febrile illnesses that are malaria (base case 8%)	3%	C, 1, 2, 3	$7,825	$1,363	$787
	20%	C, 1, 2, 3	$384	$60	$72
Probability of death for non-malarial fever given other anti-malarial (base case 0.002)	0.001	C, 1, 2, 3	$940	$141	$198
	0.05	C, 1, 2, 3	DOM	DOM	DOM
Discount rate (base case 3%)	0%	C, 1, 2, 3	$673	$106	$117
	5%	C, 1, 2, 3	$1,592	$253	$269
Probability of death for *P. falciparum* malaria given no anti-malarial (base case 0.03)	0.005	C, 1, 2, 3	DOM	DOM	$9.960
	0.04	C, 1, 2, 3	$829	$128	$149
Life expectancy in Myanmar (base case 62)	50	C, 1, 2, 3	$1,475	$234	$252
	80	C, 1, 2, 3	$974	$153	$166

*Year 1 costs are sufficient to describe the relative differences between the arms, which do not change in subsequent years. Results are based on cut-offs: 25 DALYs and/or $100 per 1,000 individuals. *Italics* = inputs that affect the order of ICERs.

**Order of ICERs based on cost, from least to most expensive. C = no intervention. 1 = arm 1, 2 = arm 2, 3 = arm 3, skipping over DOM (dominant). ***Holding other anti-malarial constant.

RDT uptake was the most significant driver of costs and DALYs. By increasing RDT uptake in each study arm from 0 to 0.65 (the highest level found in mystery client surveys), health outcomes improved significantly, and costs increased as a result of RDT use. The number of patients reached by the intervention also drove costs and health outcomes; enrollment of busy outlets receiving 40 febrile patients per month were more cost-effective than outlets seeing one febrile patient per month.

The main drivers of cost alone were programmatic staff costs and drug commodity costs. The main drivers of health outcomes alone were more diverse. Primarily, health outcomes were driven by the virulence and prevalence of febrile illnesses, shown by immense health consequences when case fatality rates of non-malarial fevers were increased (from a probability of 0.001 to 0.05). The fact that *P. falciparum* malaria was the most virulent cause of fever was also significant; while only 8% of fevers were malaria, in the absence of an RDT, health outcomes were better with higher rates (50% *vs* 3%) of presumptive treatment of fevers as malaria. Health benefits of the intervention were also proportional to malaria endemicity, shown by increasing the prevalence of malaria from 3% to 20% among febrile cases.

## Discussion

This study is the first cost-effectiveness analysis to examine behaviour change communication and incentive strategies for malaria, providing a randomized comparison of different subsidy methods with high-quality cost data. The study found that IEC was a cost-effective and promising strategy to encourage the uptake of subsidized RDTs in the Myanmar informal private sector. This strategy pays off with an attractive ICER of $200 per DALY averted as compared to subsidy with financial incentive alone (or $639 per DALY averted compared to no intervention). At base case, IEC led to four times the RDT uptake as compared to a simple subsidy (arm 1) or a subsidy with financial incentives (arm 2).

The financial incentive of offering providers one free RDT for every five RDTs purchased showed marginal improvements to a simple subsidy, with similar total costs. This strategy was therefore also cost-effective, with an ICER of $185 per DALY averted compared to a simple subsidy (or $1,159 per DALY averted compared to no intervention) although the quality of care was not as high as that which resulted from the IEC strategy. It is possible that the financial incentive used in this intervention may not be as influential as a direct cash incentive; in-depth interviews with providers revealed that the financial incentive did not motivate RDT use, and furthermore, that behaviours were mainly driven by altruism (MS, pers comm.). This finding may explain the success of the IEC strategy although other PSI interventions have suggested that providers receiving direct cash incentives were over-incentivized (PSI Myanmar staff, unpublished observations). The types of financial incentives for RDT use could, therefore, use further exploration.

Sensitivity analysis revealed three main drivers of intervention outcomes. Cost and effectiveness were driven by the number of patients that seek health care from enrolled private providers, showing the importance of targeting informal providers, the main first point of care among rural populations in Myanmar [7,12]. Costs were driven by programmatic and commodity costs, suggesting that subsidies are necessary to overcome the prohibitively high cost of malaria RDTs and ACT in the informal private sector. Health outcomes were contingent on the prevalence of non-malarial fevers and their virulence depending on treatment given, showing the importance of a confirmed non-malaria diagnosis.

Since this pilot study is meant to inform the nationwide scale-up of RDTs in Myanmar, its implications should be tailored accordingly. While costs can be predicted linearly for scale-up, programme effectiveness is much more complex. Efforts should be focused to ensure that drivers of programme effectiveness, as identified through sensitivity analysis, are optimized. As the main determinant of effectiveness was RDT uptake, monitoring and evaluation of provider adherence to the programme will be critical during scale-up. To ensure the availability of RDTs for uptake, a steady supply chain of RDTs and ACT is also essential. Programme effectiveness also depends on the number of patients reached, so busy outlets that do not enroll in the RDT subsidy scheme must be identified and targeted. Strategies to incentivize their enrollment must then be explored. Finally, the impact of this intervention is driven by the pre-eminence of the informal private sector as a point of care for fever. Care-seeking behaviour in Myanmar must continually be monitored, and in case of change, interventions will need to be redirected.

The findings in this study cannot be directly compared to other reported studies, as the cost-effectiveness of behaviour change strategies for malaria is novel. However, the implications of this study are consistent with those found in many other studies. A cost-effectiveness analysis comparing RDTs to microscopy or presumptive treatment found that in sub-Saharan Africa, RDTs were the most cost-effective strategy, an effect that increased with decreasing malaria prevalence [36]. In the present study, RDTs were also more cost-effective than presumptive diagnosis, yet cost-effectiveness was seen to increase with malaria endemicity. This was a result of high fixed programmatic costs, unaffected by growing malaria endemicity, despite increasing DALYs incurred and averted in each study arm.

In Africa, a few studies have explored the impact of providing RDT subsidies to informal providers, supporting that IEC strategies may be necessary to drive proper RDT use, which cannot be achieved by financial incentives alone. In western Kenya, despite improved RDT uptake following a simple subsidy (of 85 to 100% price reduction to the patient), the intervention was not cost-effective due to poor provider compliance to negative test results, as well as high malaria endemicity in the region (Cohen, unpublished observations). In Uganda, informal providers were trained to use subsidized RDTs, resulting in high levels of programme compliance and adherence to test results [37]. The present study has similar findings with regard to the importance of educational interventions to supplement RDT subsidies. However, it should be emphasized that behaviour change communication strategies are context specific, and that results of this study should only be applied to Myanmar.

In Myanmar, a few RDT and ACT subsidies have also been reported, showing similar implications to the study herein. A programme in 2005 entailed the provision of free RDTs and ACT to Myanmar midwives, successfully decreasing the use of artesunate monotherapy [25], and a study in 2009 showed that volunteers could be trained to use RDTs to improve malaria treatment practices [38]. A cost study on malaria treatment from 2004, which quantified patient costs for treatment-seeking within the informal private sector, found that the average malaria patient in rural Myanmar spent an equivalent of 4.2 days of per capita economic output (approximately $3.50) travelling to informal providers [39]. These high patient travel costs have an important implication to the present study, which excluded patient travel costs that would otherwise amount to $504,000 per arm annually. As patient travel costs in rural Myanmar are high, subsidized services and care should certainly be provided to alleviate total costs to the patient.

### Limitations

There are key limitations to this analysis. While the model is useful for the prediction of programmatic costs, it does not account for ongoing malaria transmission or the spread of drug resistance. Due to these limitations, there are two additional factors that must be considered for intervention scale-up. The first is coverage, as difficult-to-reach border areas with Thailand are at highest risk for artemisinin resistance [40], with agricultural migrants being the highest risk populations [41,42]. Extra care must be taken to ensure high coverage of providers in these areas, and to develop strategies to target high risk, hard-to-reach individuals [26,43]. The second factor is the sensitivity of RDTs, which limits this intervention to identification of clinical symptomatic cases of malaria only. The elimination of asymptomatic carriers, which have recently been implicated as a potentially important source of transmission of *P. falciparum* malaria [44], may therefore require additional strategies and approaches.

### Future directions

As malaria endemicity drops, it is critical to ensure that interventions keep up to date with changing epidemiology [45]. At the time of the study, only 8% of fevers in pilot study townships were due to malaria. In light of these declining rates, there are two areas of future research that must be addressed. The first is a need for a quantitative assessment of non-malarial fevers in Myanmar, and the second is to garner surveillance data from used RDTs.

A recent quantification of causes of fever in Laos, where *P. falciparum* endemicity is also declining [30], provides a valuable example Myanmar to follow. More importantly however, is a global need for strategies to manage non-malarial fevers within the informal private sector, as current practices entail the widespread use of antibiotics, threatening drug resistance [46].

The large-scale subsidy of RDTs in Myanmar also provides a potential reservoir of malaria surveillance data. Since malaria cases become clustered in low-endemic settings [44], positive RDT results should be reported to rapidly detect and eliminate pockets of infection. Dried blood spots from used RDTs can also be further analysed for molecular surveillance, as parasite DNA has shown to be easily extracted from used RDTs [47]. This can be used to detect asymptomatic carriers, and to provide insight on RDT sensitivity and quality [47].

## Conclusion

In the global fight against artemisinin resistance, the proper case management of malaria in Myanmar is critical. Malaria diagnostic and treatment practices in Myanmar can be improved by targeting subsidized RDTs and ACT to informal private providers. IEC offers a cost-effective strategy to motivate the effective use of RDTs, with further benefits offered by the addition of financial incentives to an IEC strategy, with minimal increases in cost.

## Additional file

Additional file 1:
**Cost-effectiveness analysis of malaria rapid diagnostic test incentive schemes for informal private healthcare providers in Myanmar: Supporting Information.** A detailed account of the model including a model overview, assumptions, intervention details, input data values, sources and rationale, full sensitivity analysis results, and estimated costs for recurrent years of the intervention.

## Abbreviations

ACT: Artemisinin-based combination therapy
AMTR: Artemisinin monotherapy replacement
DALY: Disability-adjusted life year
ICER: Incremental cost-effectiveness ratio
IEC: Information, education and counselling
MIS: Management information systems
PSI: Population services international
REB: Research Ethics Board
RDT: Rapid diagnostic test
WHO: World Health Organization
