# Supplementary material for: Cost-effectiveness analysis of malaria rapid diagnostic test incentive schemes for informal private healthcare providers in Myanmar
Source: Malar J. 2015 Feb 5;14:55. doi: 10.1186/s12936-015-0569-7 (PMC4334415; doi:10.1186/s12936-015-0569-7)
Supplement: Additional file 1: — Cost-effectiveness analysis of malaria rapid diagnostic test incentive schemes for informal private healthcare providers in Myanmar: Supporting Information. A detailed account of the model including a model overview, assumptions, intervention details, input data values, sources and rationale, full sensitivity analysis results, and estimated costs for recurrent years of the intervention. [file 12936_2015_569_MOESM1_ESM.pdf]

Cost-effectiveness analysis of malaria rapid diagnostic test incentive schemes for informal private healthcare providers in Myanmar: Supporting Information

Ingrid T. Chen[1], Tin Aung[2], Hnin Nwe Nwe Thant[2], May Sudhinaraset[1], James G. Kahn[1]

[1] University of California, San Francisco
[2] Population Services International, Myanmar

Table of contents

1. Decision tree overview……1
    a. Concepts……1
    b. Decision tree design……1
    c. Data inputs……2
    d. Comparison of intervention arms……2
    e. Extended dominance……3
2. Model assumptions……4
3. Intervention details……6
4. Input data sources……7
    a. Study population and epidemiology……7
    b. Malaria epidemiology and provider assessments from PSI Myanmar……7
    c. RDT pilot study data……7
    d. Cost data from PSI Myanmar……7
    e. Health outcome data……8
5. Data input values and rationale……9
    a. Malaria epidemiology and care seeking behavior……9
    b. Health outcomes……10
    c. Diagnostic test characteristics……10
    d. Provider behavior……11
    e. Rapid diagnostic test costs……13
    f. Drug costs……14
    g. Patient and provider time and travel costs……15
6. Sensitivity analysis results……17
    a. One-way sensitivity analysis for parameters that affect cost……18
    b. One-way sensitivity analysis for parameters that affect health outcomes……20
7. Results beyond first year of intervention……23
8. References……25

1. Decision Tree Overview

Concepts

We designed a decision tree using a spreadsheet in Microsoft Excel, 2010. A decision tree consists of decision nodes (denoted by squares) and chance nodes (denoted by circles). Each branch that arises from a chance branch is mutually exclusive of other branches, and the combined possibilities from each node must be collectively exhaustive such that branch probabilities add to 1. A decision tree that compares health service delivery options conventionally starts with each incentive method denoted as a decision option.

The decision tree is a flow chart that aims to encompass all possible courses of action within a chosen population (Figure 1). The population for this study comprises of febrile patients in six townships within Myanmar's Mon and Shan states that seek healthcare within the informal private sector from May to September. By following the decision tree from left to right, each pathway of action is represented by a terminal node (denoted '//'). The decision node represents subsidy schemes while chance nodes are categorized as: disease state, provider behavior, diagnosis, and health outcomes.

Figure 1. Decision tree model for malaria RDT subsidy schemes

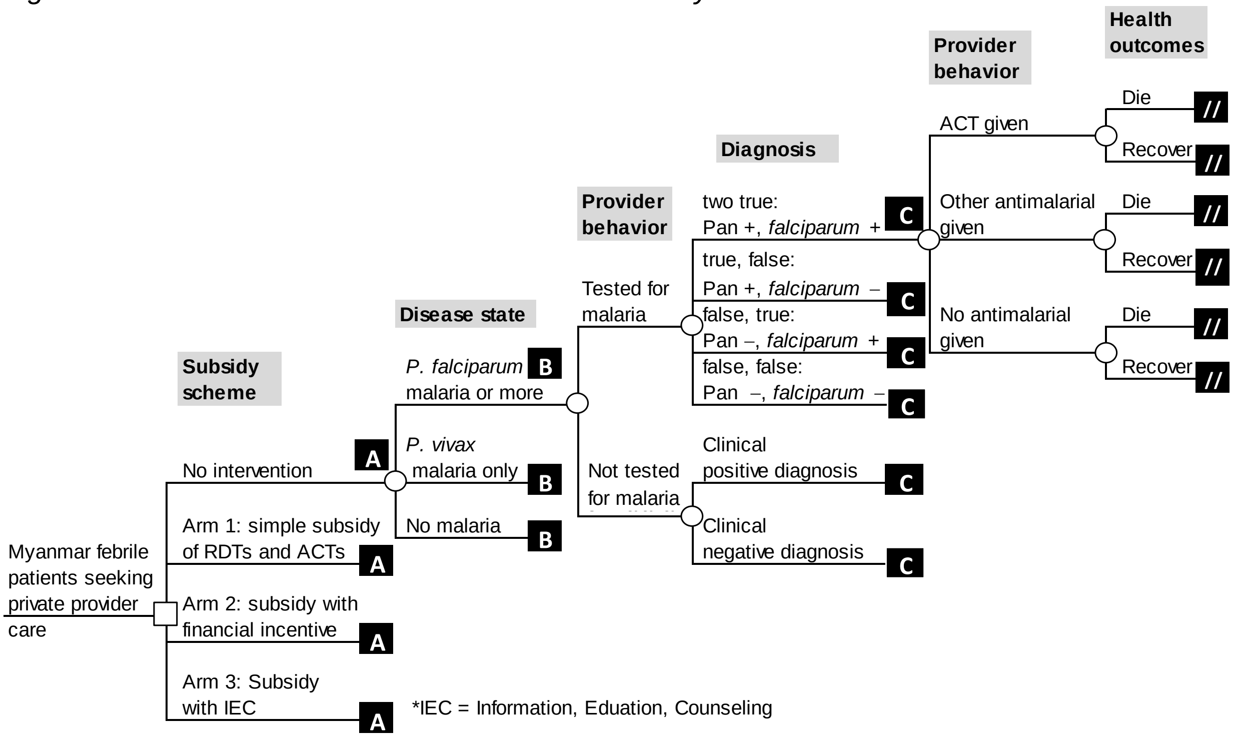

Decision Tree Design:

In the beginning of the decision tree, each subsidy method is represented as a decision We use 'no intervention' as a base case for reference. For each subsidy scheme, we use a conventional decision tree structure for diagnostic tests, which starts with the true disease state as the first chance node. Our disease states are: *P. falciparum* malaria or more (including *P. vivax*), *P. vivax* malaria only, and no malaria, as we assume that all non-*falciparum* malaria is *P. vivax* malaria.

For each disease state, we then use a chance node for whether the provider uses an RDT. If the patient is tested with an RDT, there will be four possible test results for the combined pan *Plasmodium* test and *P. falciparum* diagnostic test: ++, +-, -+, and --. The probability of each result depends on whether the test result is true or false: a true positive corresponds to test sensitivity, false negative to (1 – sensitivity), true negative to specificity, and false positive to (1 – specificity). If the provider does not use an RDT, the resulting clinical diagnosis will be positive or negative for malaria. We assume that the pan *Plasmodium* test and

P. falciparum test performance is independent: failure to detect malaria with the pan *Plasmodium* PLDH antigen-detecting test is uncorrelated with failure to detect malaria with the *P. falciparum* HRP2 antigen-detecting test.

Each test result then leads to one of three treatment possibilities that are chosen by the provider. The provider sells subsidized ACTs, other antimalarials, or no antimalarial to the patient. After each treatment, the patient either lives or dies, which is a terminal node represented by "//". If the patient lives, morbidity associated with illness is accounted for in quantified health outcomes.

Data Inputs

There are three types of data inputs described below. The actual sources of data are described in section 4 (Data Inputs).

Probability Data

A probability value is assigned to each node. Each decision node is assigned a probability of 1, and each chance node is assigned a total probability of 1. Since chance nodes encompass a mutually exclusive and collectively exhaustive set of possible actions, the sum of probabilities that emerge from each chance node adds to 1.

The probability of occurrence for each path leading to a terminal node is calculated as the product of probabilities of every chance node from the decision node to that terminal node. The probability of occurrence is used to weight the associated costs (measured in USD) and health outcomes (measured in DALYs) of each path.

Cost Data

Cost data is incorporated at every applicable node and measured in USD. The exchange rate used is 907 Kyats / dollar, the official rate on May 1st 2013. Exchange rates in the model can be changed and updated easily. The total cost associated with each path is weighted by probability, and the sum of weighted costs for each decision node is the total cost for that decision node.

Health Outcome Data

The health outcome that corresponds to every terminal node is quantified in DALYs. Life years lost to mortality are calculated by subtracting the mean life expectancy from the average age of malaria-induced death and applying a 3% discount rate to each year in the future. Discounting is typically used in CEA to account for the fact that people tend to value events in the present more than in the distant future. For surviving patients, the morbidity of those who do not recover immediately will be calculated by dividing the average duration of illness by the DALY weight for the illness. The health outcomes associated with each path are weighted by probability, and their sum for each decision node represent the total DALYs incurred for that decision node.

Comparison of Intervention Arms

Once probabilities, weighted costs, and weighted health outcomes are established, the sum of weighted costs and health outcomes for each decision node (subsidy scheme) are used to compare the three intervention arms and the base case (no intervention). We compare the subsidy methods by ordering the approaches from least to most expensive. The subsidy methods were first obtained incrementally. The incremental cost-effectiveness ratio (ICER) for each intervention is obtained by the following formula:

$$\text{ICER} = \frac{\text{(Cost of intervention – cost of next less expensive approach)}}{\text{(DALYs with intervention) – (DALYs with next less expensive approach)}}$$

Extended dominance

In this study, we found that increasing programmatic interventions led to increases in costs that were compensated by larger increases in effects. This led to decreasing ICERs. This is called "extended dominance", where a combination of ‘no intervention’ and the last intervention (arm 3) is more cost-effective than the intermediate interventions (arm 1). The proper comparison is thus each intervention versus the reference, not versus the next-least expensive option. The formula used in this case:

$$\text{Cost per DALY averted vs baseline} = \frac{(\text{Cost of intervention} - \text{cost of no intervention})}{(\text{DALYs with intervention}) - (\text{DALYs from no intervention})}$$

2. Model assumptions

The purpose of this study is to inform policy-makers on scale-up methods, so we deliberately do not account for malaria transmission (from untreated cases) or the likelihood of selection of resistance in this model. The model applied the following assumptions based on the rationale detailed below.

1. Artemisinin monotherapy is crowded out by quality-assured ACTs.
Rationale: During the Artemisinin Monotherapy Replacement (AMTR) program mystery client survey in late 2012 which recorded fever drug stocks four months after ACT rollout, only 4.3% provider-recommended fever drugs were artemisinin monotherapies. By contrast, 54.1% of provider-recommended fever drugs were quality-assured ACTs. The replacement of artemisinin monotherapies with ACTs is well underway, and monotherapies in Myanmar have been banned since December 31st 2012.[1] The 2013 mystery client survey affirms that no artemisinin monotherapies were prescribed to any mystery clients. Qualitative demographics also showed that very few outlets still stocked monotherapies (2/31, who were also selling ACTs).

2. All medicine is of high quality: there are no counterfeits or expired drugs.
Rationale: artemisinins are the most common counterfeit durgs,[17] and all studied providers are receiving subsidized quality-assured ACTs. We validated this assumption during interviews with private providers, where all outlets had the subsidized Supa Arte product in stock, none of which had expired.

3. The subsidized RDTs are stored properly and can be accurately characterized by reported sensitivity and specificity measurements.[2]
Rationale: Although high heat or humidity can compromise the quality of RDTs. Intervention provider training sessions emphasized how to properly store RDTs and to check for expiration dates. Interviews with private providers will also check the expiration dates of any RDTs in stock.

4. ACTs and RDTs are distributed through the same channels as all medicines considered in the decision tree model: antipyretics, antibiotics, and non-artemisinin antimalarial monotherapies.
Rationale: there is no data available to track the distribution of other products within the informal private sector. We used estimates provided by program staff leaders at PSI Myanmar to predict price mark-ups between from wholesale to retail to provider to the patient.

5. Either P. falciparum or P. vivax malaria is present in all malaria infections.
Rationale: *P. malariae* and *P. ovale* parasites are technically difficult to differentiate from *P. vivax* parasites. WHO reports on the malaria burden in Myanmar do not account for *P. malariae* and *P. ovale* because they are considered to be rare, and furthermore can be treated with therapeutics that effectively treat *P. vivax* malaria. Both *P. malariae* and *P. ovale* parasites can be detected by the Pan *plasmodium* RDT test so even if these parasites are present without *P. vivax* infection, the model results will be unaffected. A report in 1998 showed that the prevalence of *P. malariae* at the Thai-Myanmar border was 24.3%, which is much higher than the Myanmar department of health estimate of 0.1%.[3]

6. Subsidized ACTs are sold only as a full course of therapy.
Rationale: the 2012 mystery client survey for the AMTR project shows that 97% of quality-assured ACTs were sold as full courses of therapy. This fact attests to the success of the AMTR project strategy to discourage providers from cutting the blister packets of subsidized ACTs. Providers were previously known to cut blister packets of artemisinin monotherapy prior to sale, so the AMTR package design team intentionally nested the blister packet in a cardboard envelope that was sealed with a sticker, making it very difficult to cut the full course of ACTs into pieces. Results from the 2013 Mystery Client survey in the pilot study affirm that all courses of ACT prescribed were full courses.

7. Patients adhere to a full 3-day course of subsidized ACTs.

Rationale: the AMTR project uses a multipronged approach to encourage the completion of three-day ACT regimens: 1) the price of a full course of ACTs is set to match the price of partial courses of artemisinin monotherapy that patients afford 2) both provider support visits and community outreach programs emphasize the importance of completing a full course of ACTs 3) the design of the ACT packaging includes two written Burmese reminders to complete a full course of ACTs: one on the front of the cardboard envelope and a second below the pills inside the envelope. The RDT household surveys will confirm whether this assumption is accurate: otherwise the model will be updated accordingly.

8. 'Other antimalarial' refers to the use of quinine or chloroquine.

Rationale: The 2012 AMTR mystery client survey showed that only 8% of fever diagnoses were treated with non-artemisinin antimalarials. We chose quinine or chloroquine based on in-depth interview stock audit data from the pilot study, triangulated with conversation with PSI Myanmar program staff. Interestingly, none of the providers screened carried primaquine, which is the only drug combination capable of clearing hypnozoites, the latent liver stage of *P. vivax* infections.

9. Drug adherence to chloroquine and injectable quinine is high given the short course of therapy.

Rationale: Consultation with PSI Myanmar program officers suggested that patients seeking private sector care typically adhere to the first 2-3 days of a drug regimen. Pilot study qualitative demographic data shows that most providers who carry quinine carry it as an injectable solution. The injection is available as a single dose, and orally administered chloroquine is available as a three-day course of therapy.

10. P. vivax malaria does not relapse.

Rationale: The complexity of relapse and unavailability of epidemiological data prevent the accurate prediction of *P. vivax* relapse. A full course of primaquine is required to ensure the clearance of hypnozoites, the parasite stage responsible for the relapse of *P. vivax* malaria. Relapse rates depend on the duration of fever before initial treatment, the type of treatment used, the level of parasitemia, and the level of patient drug adherence.[4] Relapse rates are also likely to be low within the 1-month time frame considered by the model: a study at the Thai-Myanmar border showed a 28-day relapse rate of 3.4% for self-administered therapy and 0% for directly-observed and primaquine therapy.[4]

12. 'No antimalarial' refers to the use of antipyretics 70% of the time and antibiotics 30% of the time.

Rationale: A 2012 mystery client survey at PSI Myanmar showed that when antimalarials were not prescribed for fevers, 50% of cases were treated with antibiotics and the other 50% with antipyretics. However, more recent household surveys at PSI Myanmar showed that the vast majority (90% estimated) of providers administered antipyretics to patients presenting with a fever. We therefore assume that patients receive antipyretics 70% of the time.

13. Only one type of medication is prescribed at any given time.

Rationale: While some providers in Myanmar are known to administer "machine gun therapy" by prescribing multiple drugs, the 2012 PSI mystery client survey shows that only 0.4% of providers administered more than one drug at a given time.

3. Intervention details

The pilot study took place from May to September, 2013. The 631 outlets enrolled were in the RDT pilot study (Table 1). The pilot study townships are shown in the map in Figure 2, of which Artemisinin Monotherapy Replacement (AMTR) outlets targeted for RDT scaleup are shown in red.

Table 1. Outlets reached by RDT pilot study.

State	Township	Intervention arm
Mon	Paung	Arm 1: simple subsidy
Mon	Bi Lin	Arm 2: subsidy with financial incentive
Mon	Than Byu Za Yat	Arm 3: subsidy with Information, education and counseling (IEC)
Shan	Mongpyak	Arm 1: simple subsidy
Shan	Nam Kam	Arm 2: subsidy with financial incentive
Shan	Hseni	Arm 3: subsidy with IEC
Total outlets enrolled	631	

Figure 2. Map of Myanmar AMTR project sites with RDT project offices and townships

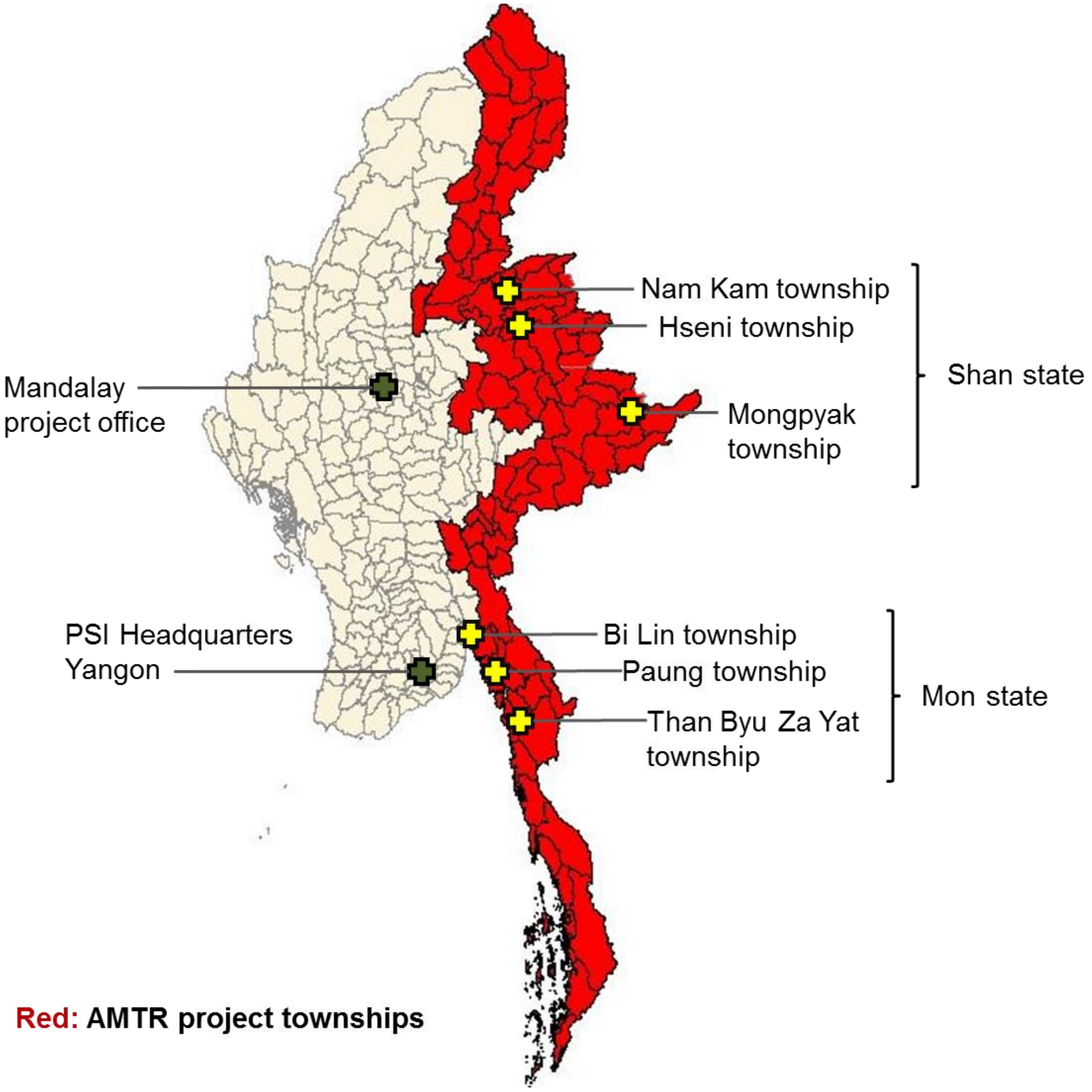

4. Input data sources

A combination of finance/account records and management information systems (MIS) data from PSI Myanmar, mixed methods data from the RDT pilot study (household surveys, interviews with private providers, mystery client visits, stock audit data from supply points), and a review of published scientific literature were used as detailed below.

Study Population and Epidemiology

The study population was defined as the number of patients that seek private provider care at all outlets enrolled in the RDT pilot study from May to September, 2013. Study population data was derived from malaria epidemiology and provider assessments from other PSI Myanmar programs and a review of published scientific literature.

Malaria Epidemiology and Provider Assessments from PSI Myanmar

PSI Myanmar has detailed Management Information System (MIS) data on malaria RDT uptake as well as provider-reported RDT test results and patient demographics. The data is a part of the PSI Sun Primary Health (SPH) social franchising brand for private providers throughout rural Myanmar.[5] We also used SPH monitoring mechanisms to estimate baseline provider knowledge levels of RDT use. A patient simulation known as the Sustained Quality study in 2011 used direct observation, clinical vignettes, and medical mannequins to assess provider understandings of malaria diagnosis and treatment before and after a single RDT training session (time points: 6 and 12 months post training). The study assessed provider knowledge levels in the following categories: medical history taking, looking for signs of severe malaria, checking for vital signs, malaria drug history, RDT performance, proper referral to higher level facilities, and drug prescription and information.

RDT Pilot Study Data

The RDT pilot study used four analytical methods: 1) household surveys, 2) interviews with private providers, 3) mystery client visits, and 4) stock audit data from supply points. Each method was used to evaluate RDT uptake and intervention effectiveness. The household surveys measured RDT uptake at a community / patient level before and after the intervention. The interviews with private providers explored provider attitudes to RDT use at the end of the pilot study and also included a survey of antimalarial and RDT stock and prices. Mystery clients presented with an alleged fever to providers that are enrolled in the pilot study as an additional measure for RDT uptake in the beginning and end of the pilot study. Stock audit data monitored RDT and ACT use, which was possible through the program requirement that used RDTs be returned to supply points for resupply. Providers were instructed to record the date, result of the test, and patient age / gender on each test. A comparison of RDT results with ACT sales suggested whether ACTs are being sold as recommended (for *P. falciparum* malaria only).

Cost Data from PSI Myanmar

Cost data was derived from PSI Myanmar finance and account records, and the exchange rate used is 907 Kyats / dollar, the official rate on May 1st 2013.[6] Cost-effectiveness analysis from a societal perspective included operational costs to PSI Myanmar, commodity costs across the supply chain, as well as time and commodity costs to the patient and provider.

Operational costs were informed by PSI Myanmar finance and account data. To allow for the prediction of long-term operational costs, we defined operational costs in two categories: the cost of program initiation and the cost of recurrent program activities.

Program initiation consisted of staff training sessions, provider recruitment activities, and community education sessions on the utility and availability of subsidized RDTs. Community education sessions were considered to be a program initiation cost because once all intervention areas were reached, community education was considered complete and sessions were no longer continued. PSI program staff members expect that for scale-up, six months of community education sessions are sufficient to reach communities throughout the intervention

areas. Staff training sessions are also a program initiation cost: PSI training program designers expect the initial staff training session to be much more involved and costly than additional refresher training sessions which take place every 6-12 months. Refresher training costs were not included in this analysis. The costs of research and preparation required for program initiation were also excluded.

Recurrent program activities included all field activities and office support required for program rollout. Field activities included the management and delivery of provider visits and community education sessions, and office support included administrative and managerial staff members. We excluded the cost of pilot study evaluative methods (i.e. mystery client visits) since research evaluative methods do not represent scale-up practices.

For both program initiation and recurrent program activities, the RDT pilot study had three types of operational costs: 1) overhead costs for PSI Myanmar, 2) staff costs, and 3) program materials and supplies. All costs were scaled to estimate the monthly operational costs for each arm of the RDT pilot study.

Commodity costs were mapped across the supply chain, starting from manufacturing and ending at the patient's drug or RDT purchase (Figure 2). Costs included product costs and delivery costs. RDT costs from manufacturing to pharmacy supply point delivery were recurrent operational costs. RDT purchases from pharmacy supply points to private outlets are provider costs, and individual RDT purchases from private informal outlets are patient costs.

Figure 2. Malaria RDT supply chain for RDT pilot study

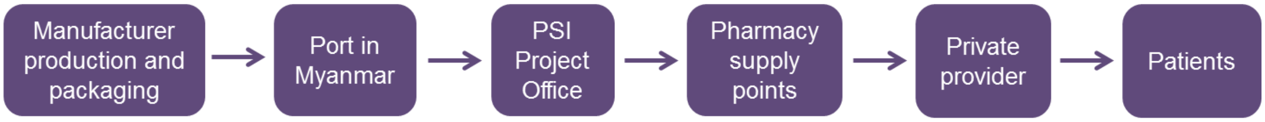

Patient and provider commodity costs were estimated through data from PSI programmatic activities as well as staff consultation. There were two sources of baseline data that are available from the AMTR program evaluation in late 2012: a full stock audit and a mystery client survey. The RDT program is nested within the AMTR program, which has been delivering subsidized ACTs to 6,865 private outlets throughout eastern Myanmar since September 2012. The AMTR stock audit reached 1,159 private outlets that were enrolled to purchase subsidized ACTs, recording all available antimalarials and RDTs. The mystery client survey reached 446 private providers to establish baseline provider behavior for patients that present with fever. The mystery client survey also recorded antimalarial and RDT stock and prices, the proportion of providers that used RDTs, the drugs recommended for sale to the patient, and the level of provider instruction to the patient on ACT use. Patient and provider time costs were estimated through informal discussion with PSI Myanmar staff members. Patient traveling costs are estimated from a similar CEA in scientific literature. We excluded the potential lost income from healthcare seeking and illness due to the unavailability of data.

<u>Health Outcome Data</u>

Health outcomes were quantified in Disability Adjusted Life Years (DALYs), with a 3% discount rate applied.

5. Data Input values and rationale

Table 2. Malaria epidemiology and care-seeking behavior

Subject	Input value	Source(s) and/or rationale
Percentage of *P. falciparum* / *P. vivax* malaria	65% *falciparum*<br><br>35% *vivax*	Reference 7, WHO SEARO data: 70% *P. falciparum* in 2006.
		National Malaria Control Program estimate in August 2012 from PSI Myanmar: 68%
		PSI Myanmar program staff: *falciparum* rates have declined to nearly 60% in the Mon state due to high NGO presence
		Pilot study stock audit data: 55% of returned malaria positive RDTs showed *P. falciparum* and mixed Plasmodium infections, while 45% of these RDTs showed *P. vivax* only.**
		Estimate based on the above: 65%
Proportion of febrile cases in population that are malaria	8%	PSI Myanmar MIS data:* 7.2%<br>Pilot study stock audit data: 8.56% of fever cases tested were malaria according to returned RDTs**
Average number of febrile patients that visit one private provider per month	20	PSI Myanmar MIS data estimated 20 per month.
		Pilot study stock audit data showed that 1-4 RDTs were used by each provider per month. Baseline uptake levels are between 9-16% (Table 6), therefore the estimated number of clients is between 1/16% and 4/9%: 6 to 44.

* MIS data is from SPH interventions from July to October 2012 in the same Mon state townships as the RDT study. The sample includes 3769 patients that were tested for malaria within 24 hours of the onset of fever. MIS data from the Shan state was not available.

** Returned RDTs were both read by providers (the results were recorded on the RDT using a black permanent marker) as well as PSI staff. The reads between provider reports and PSI staff showed high concordance, and we chose to use provider reports since the rate of false positive RDT test results increases past the recommended 20-minute readout.

Table 3. Case fatality rates

Subject		Value	Source(s) and/or rationale
Case fatality rates for *P. falciparum* malaria*	Given ACT	0.0001	Very low probability
	Given chloroquine or quinine	0.007	Assumption because of high rates of chloroquine resistance[8]
	Given no antimalarial	0.03	Ref. 9, hospital case fatality rate in Bago Myanmar: 2.7% for uncomplicated *P. falciparum* malaria, 22% for cerebral. Ref. 10: 3% case fatality rate for *P. falciparum* malaria on eastern border of Myanmar.
Case fatality rates for *P. vivax**	Given ACT	0.0001	Very low probability: blood-stage parasites cleared
	Given chloroquine or quinine	0.0001	Ref. 11, Published materials on *P. vivax* treatment rates with chloroquine in Papua.
	Given no antimalarial	0.01	Ref.12: the case fatality rate for multidrug resistant *P. vivax* malaria in Papua was 1.4%. We estimate a slightly lower rate because patients can seek retreatment for drug sensitive *P. vivax* malaria.
Case fatality rate for non-malarial febrile illnesses*	Given ACT or other antimalarial	0.002	Ref. 13: mortality analysis from hospital and village records in Bago, Myanmar. 40% of febrile deaths are non-malarial. Triangulated with PSI MIS data from Bago: 8% of fevers are malaria. Malaria is 17.5x more deadly than other fevers.

	Given no antimalarial	0.0016	Ref. 14: WHO burden of disease in Myanmar: categorized febrile illnesses treatability with antibiotics in appendix B to estimate 2/3 nonmalarial fevers are treatable with antibiotics.<br>Ref. 15: confirms that a large proportion of non-malarial fevers in neighboring country Laos are treatable with antibiotics.<br>Assumption 12, 30% of no antimalarial administration is an antibiotic.<br>We estimate that 2/3*30% (= 20%) of nonmalarial fevers get treated properly, the remaining 80% suffer the same fatality rate as those given ACT or other antimalarial.

Table 4. Health outcomes

Subject		Value	Source and Comments
**Survival**			
Average duration of malaria illness without effective treatment		1 week	Ref. 16: hospital-based records indicate that most individuals check into the hospital 5-8 days of malarial illness with signs of severe malaria.
DALY weight of malaria		0.2	On a scale of 0 to 1: ref. 17.
Average duration of non-malarial febrile illness		1 week	Assumption
DALY weight of non-malarial fever		0.18	Estimate based on Ref. 18, Global Burden of Disease: infectious diseases assigned 0.21 DALY weight for acute, 0.053 for moderate.
**Mortality**			
Mean life expectancy in Myanmar		62	Took the average of three data points:<br>64.7: Ref. 19, World Bank data.<br>56: Ref. 20, Global Burden of Disease.<br>64.2: Ref. 21, Global Burden of Disease.
Average age of malaria-induced death in intervention townships*		25	MIS data from PSI Myanmar from 374 confirmed positive malaria cases.
DALYs incurred malaria death	No discount rate	38.00	Calculated as years of life lost - (DALY weight of malaria * 1 week of illness)
	3% discount rate	22.82	
Average age of non-malarial febrile death in Myanmar		30	MIS data from 4,853 confirmed negative malaria cases.
DALYs incurred non-malarial fever death	No discount rate	33.00	Calculated as years of life lost - (DALY weight of non-malarial fever * 1 week of illness)
	3% discount rate	21.07	

*Data only available from intervention townships in the Mon state

Table 5. Diagnostic Test Characteristics

Subject		Value	Source(s) and/or rationale
RDT sensitivity and specificity	*P. falciparum* sensitivity	100% (FR and SD)	Ref. 2: Foundation for Innovative New Diagnostics (FIND) WHO RDT data, at 200 parasites / μL.<br><br>SD = Standard Diagnostics Ag Pf Pv, Korea. Given during the first 2 months of the RDT pilot study.<br><br>FR = First response Pf Pan from Premier Medical Corporation, India. Given during the last 4 months of the RDT pilot study.
	P. falciparum specificity	97% (FR and SD)	
	Pan *plasmodium* sensitivity	92% (estimate)<br><br>100% (SD)<br><br>88% (FR)	
	Pan *plasmodium* specificity	98% (Estimate)	

		95% (SD)<br><br>100% (FR)	
Clinical diagnostic result	Clinical positive diagnosis	0.50	The data used does not indicate whether the provider believes if the patient has malaria or not. Therefore, the corresponding prescription probabilities for clinical positive and negative diagnoses are the same. The values in this field do not affect results and we use a provisional 50% probability of positive or negative diagnosis.<br><br>Actual values likely range between 24 and 82% according to mystery client surveys.<br><br>At baseline, in 2012, 82% of mystery clients receiving clinical diagnosis were treated with ACT or other antimalarials. However, the 2013 mystery client survey only showed 24-37% of individuals received ACTs or antimalarials for alleged fever.
	Clinical negative diagnosis	0.50	

Table 6. Provider behavior (Bolded numbers are used as inputs)

**Subject**		**Probability**				**Source**
		**No intervention**	**Arm 1**	**Arm 2**	**Arm 3**	
**Diagnostic method**						
Clinical diagnosis		**0.98** (HH)<br>0.89 (MC 2012)	**0.98** (HH)<br>49.2 (MC 2013)	**0.98** (HH)<br>36.4 (MC 2013)	**0.92** (HH)<br>41.5 (MC 2013)	Baseline: 2012 mystery client survey.[+]<br><br>HH survey (weighted data, denominator only includes private informal provider types in study).
RDT		**0.02** (HH, n = 1)*<br>0.11 (MC 2012)	**0.02** (HH, n = 0)*<br>50.8 (MC 2013)	**0.02** (HH, N = 0)*<br>63.6 (MC 2013)	**0.08** (HH)<br>58.5 (MC 2013)	
**Prescription**						
Diagnosis	Medicine prescribed					
Clinical + malaria	ACT	**0.05**<br>0.7 (MC 2012)****<br>0 (HH) n=0	**0.12**<br>0.107 (MC 2013)<br>0.13 (HH) n=16	**0.12**<br>0.105 (MC 2013)<br>[0.13]***<br>(HH) n=0	**0.19**<br>0.25 (MC 2013)<br>[0.13]***<br>(HH) n=0	2012 Mystery client survey**<br>2013 Mystery client survey: clients do not have malaria.<br>Value used: an average between 2013 mystery client and household survey data.
	Other antimalarial	**0.03**<br>0.06 (MC 2012)<br>0.003 (HH) n=1	**0.07**<br>0.14 (MC 2013)<br>0.008 (HH) n=1	**0.07**<br>0.11 (MC 2013)<br>0.009 (HH) n=1	**0.07**<br>0.13 (MC 2013)<br>0.014 (HH) n=2	
	No antimalarial	**0.92**<br>0.24 (MC 2012)<br>0.992 (HH) n=383	**0.81**<br>0.753 (MC 2013) 0.992 (HH) n=119	**0.81**<br>0.879 (MC 2013) 0.991 (HH) n=107	**0.74**<br>0.625 (MC 2013)<br>0.986 (HH) n=139	

Clinical - malaria	ACT	**0.05**<br>0.7 (MC 2012)<br>0 (HH) n=0	**0.12**<br>0.107 (MC 2013)<br>0.13 (HH) n=16	**0.12**<br>0.105 (MC 2013)<br>[0.13] (HH) n=0	**0.19**<br>0.25 (MC 2013)<br>[0.13] (HH) n=0	
	Other antimalarial	**0.03**<br>0.06 (MC 2012)<br>0.003 (HH) n=1	**0.07**<br>0.14 (MC 2013)<br>0.008 (HH) n=1	**0.07**<br>0.11(MC 2013)<br>0.009 (HH) n=1	**0.07**<br>0.13 (MC 2013)<br>0.014 (HH) n=2	
	No antimalarial	**0.92**<br>0.24 (MC 2012)<br>0.992 (HH) n=383	**0.81**<br>0.753 (MC 2013)<br>0.992 (HH) n=119	**0.81**<br>0.879 (MC 2013)<br>0.991 (HH) n=107	**0.74**<br>0.625 (MC 2013)<br>0.986 (HH) n=139	
RDT Pan + *falciparum* +	ACT	**0.75** (SPH)<br>0 (HH) n=0	(HH n=0)<br>0.857 (MC 2013)<br>Correct treatment MC = 0.857 * 0.914 = **0.78**	(HH n=0)<br>0.972 (MC 2013)<br>Correct treatment MC = 0.972 * 0.861 = **0.84**	(HH n=0)<br>0.978 (MC 2013)<br>Correct treatment MC = 0.978 * 0.889 = **0.87**	Prescriptions for 'no intervention' are informed by the PSI SPH patient simulation assessment, 2011, estimated for arms 1-3.
	Other antimalarial	**0.05** (SPH)<br>0.50 (HH) n=1	(HH n=0)<br>**0.05**	(HH n=0)<br>**0.05**	(HH n=0)<br>**0.05**	
	No antimalarial	**0.2** (SPH)<br>0.50 (HH) n=1	(HH n=0)<br>**0.17**	(HH n=0)<br>**0.11**	(HH n=0)<br>**0.08**	
RDT Pan + *falciparum* -	ACT	**0.5**<br>0.6 (SPH)<br>1.0 (HH) n=2	**0.10**<br>1.0 (HH n=1)	**0.10**<br>(HH n=0)	**0.10**<br>(HH n=0)	Qualitative interviews show that typically other antimalarials or antibiotics are given for vivax malaria.
	Other antimalarial	**0.25**<br>0.2 (SPH)<br>(HH n=0)	**0.45**<br>(HH n=0)	**0.45**<br>(HH n=0)	**0.45**<br>(HH n=0)	
	No antimalarial	**0.25**<br>0.2 (SPH)<br>(HH n=0)	**0.45**<br>(HH n=0)	**0.45**<br>(HH n=0)	**0.45**<br>1.0 (HH) n = 1	
RDT Pan - *falciparum* +	ACT	**0.75**<br>(SPH)<br>0 (HH) n=0	(HH n=0)<br>0.857 (MC 2013)<br>Correct treatment MC = 0.857 * 0.914 = **0.78**	(HH n=0)<br>0.972 (MC 2013)<br>Correct treatment MC = 0.972 * 0.861 = **0.84**	(HH n=0)<br>0.978 (MC 2013)<br>Correct treatment MC = 0.978 * 0.889 = **0.87**	Assuming same prescriptive behavior as Pan + *falciparum* +, as qualitative in-depth interviews show that providers understand that *falciparum* + should be treated with ACTs
	Other antimalarial	**0.05** (SPH)<br>0.50 (HH) n=1	(HH n=0)<br>**0.05**	(HH n=0)<br>**0.05**	(HH n=0)<br>**0.05**	
	No antimalarial	**0.2** (SPH)<br>0.50 (HH) n=1	(HH n=0)<br>**0.17**	(HH n=0)<br>**0.11**	(HH n=0)<br>**0.08**	
RDT Pan - *falciparum* -	ACT	**0.4** (SPH)<br>(HH n=0)	**0.057** (MC)<br>(HH n=0)	**0.083** (MC)<br>(HH n=0)	**0.022** (MC)<br>(HH n=0)	Results taken directly from mystery client providers that used RDTs.
	Other antimalarial	**0.02** (SPH)<br>(HH n=0)	**0.029** (MC)<br>(HH n=0)	**0.056** (MC)<br>(HH n=0)	**0.089** (MC)<br>(HH n=0)	
	No antimalarial	**0.58** (SPH)<br>1.0 (HH n=8)	**0.914** (MC)<br>1.0 (HH n = 4)	**0.861** (MC)<br>1.0 (HH n = 12)	**0.889** (MC)<br>1.0 (HH n = 13)	

* Numbers adjusted to be the same as no intervention due to small sample sizes
** 70% use of ACTs is an average value between the Mon and Shan state results from the 2012 mystery client survey. We scale the mystery client prescription of other antimalarials and no antimalarial (antibiotic and antipyretic) to a 70% ACT uptake.
*** Numbers adjusted to match arm 1 since no data available for arms 2 and 3
**** Lowered estimate of actual ACT use because the 2012 mystery client survey took place 4 months after ACTs were first introduced, possibly leading to temporary overuse.

RDT Costs

RDT commodity costs include the cost of subsidy and are separated as costs to the donor, provider and patient (Table 6). The RDT commodities used are: Standard Diagnostics (SD) Bioline Ag Pf Pv from Korea (used in the first 2 months of the intervention) and First Response, Premier Medical Corporation from India (used in the last 4 months of the intervention). The cost of RDT distribution across the supply chain is detailed in Table 7. Calculations for RDT costs are based on product markup costs between distribution stages, estimated to be: 3% from wholesale to retail, 20% from retail to distributor, and 100% from vendor to patient based on discussion with PSI program staff managers (Table 8). Estimates are indicated by red text while hard numbers are denoted by black text.

Table 6. RDT costs from a societal perspective

Perspective	No intervention	Arm 1	Arm 2	Arm 3
Societal*	$1.16	$0.68	$0.80	$0.68
Donor	$0.00	$0.36	$0.48	$0.36
Provider	$0.48	$0.11	$0.09	$0.11
Patient	$1.16	$0.32	$0.32	$0.32

*Societal = donor costs + patient costs.

Table 7. RDT distribution across the supply chain

**Distribution stage**	**Delivery Cost (USD)**	**Comments**
Manufacturer to PSI headquarters	$0.01 per unit	$3,269 for 400,000 units. Accounted for in RDT unit costs.
PSI headquarters to project office	$61.77 per month	Accounted for in recurrent program costs
PSI project office to supply point	N/A	For the pilot study, RDTs were transported in the backpack of Jr. Health Service Officers.
Supply point to private provider	$11.02 per provider per month	For the pilot study, resupply was done by both Product Promoters and providers. Stock audit data showed that 22 – 47% of RDTs sold were returned. Accounted for in provider travel costs and provider time costs.
Provider to patient	$3.50 per patient	Patient travel estimates were not included in the analysis (estimate from reference 19).

Table 8. RDT cost calculations across the supply chain

	**Cost at Level of Distribution (USD)**						**Source / triangulated data**
	**Wholesale***			**Retail**	**Vendor**	**Consu-mer**	
	**Cost per package**	**Shipping**	**Total**				
No interven-tion	$0.47	$0.01	$0.48	$0.48	$0.58	$1.16	Cost per RDT kit is $0.41, cost per pair of gloves is $0.06. Field staff estimated consumer price $1.10 Qualitative demographics: average price to providers, $0.11, consumer price: $0.30. MC survey: average cost of RDT was $0.35
Arms 1 or 3	$0.47	$0.01	$0.48	$0.12	$0.18	$0.32	
Arm 2**	$0.56	$0.01	$0.57	$0.09	$0.15	$0.32	

* Cost per packet includes cost per unit, packaging and labor.
** Arm 2 prices reflect that for every 5 RDTs purchased at retail, the donor has to provide 1 for free.

Drug Costs

Table 9 summarizes drug costs: when the cost of marketed drugs could not be obtained, we use wholesale drug prices to estimate the cost to the patient. Product markup costs between distribution stages are estimated to be: 3% from wholesale to retail, 20% from retail to distributor, and 67% from vendor to patient based on estimates from PSI program staff managers. Unsubsidized drugs are considered to be patient costs while the donor cost of subsidized ACTs is also calculated (Table 10).

Table 9. Drug Costs

Commodity	Patient Cost	Source
ACT	$0.53	Table 10, Provider in-depth interview demographic data, Mystery Client Survey 2012, discussion with PSI Program manager
Quinine and chloroquine	$0.55	Quinine and chloroquine prices based on in-depth interview demographics and mystery client survey. No prices given for chloroquine, quinine costs $1.65. Mystery clients paid between $0.22 to $1.76. The mode in the mystery client was $0.55
Antibiotics^{+}	$0.93	Table 11: antibiotic prices based on wholesale costs from PSI social franchising account records
Antipyretics	$0.44	Personal purchase of 8 paracetemol tablets in the Shan state.
Cost of “no antimalarial” calculation	$0.58	Table 11: calculated according to 70% administration of antipyretics and 30% of antibiotics

Subsidized ACTs are sold as four products: Supa Arte 1, 2, 3 and 4. The Supa Arte products, marketed from Ipca laboratories limited in India, contain various doses of artemether and lumefantrine targeting different age ranges. We scaled the percentage of use for each Supa Arte product according to the percentage of individuals within each age range that were tested for malaria in the Mon state from July to October, 2012 to obtain a weighted average cost of an ACT (Table 10). The exchange rate used is the rate at the time of purchase: 845.94 Kyat/dollar.

Table 10. Calculation of average Supa Arte cost to intervention patients

Supa Arte Product	Age range	# of tablets*	Wholesale price (USD)	Percentage of malaria patients in age range **	Weighted costs per patient on average
1	N/A	N/A	$0.53	6.3%	$0.03
2	N/A	N/A	$0.95	22.2%	$0.21
3	N/A	N/A	$1.36	16.8%	$0.23
4	N/A	N/A	$1.70	54.7%	$0.93
Total					**$1.40**

* Each tablet contains 20 mg of artemether and 120 mg of lumefantrine.
** Based on percentage of RDTs used by SPH providers in the Mon State from July-October, 2012.

To obtain societal drug costs, consumer or unit costs were obtained where available. Markup rates throughout distribution were used to estimate societal costs (Table 11). Wholesale costs include the cost of commodity, packaging materials, and packaging labor. All costs up to the retail level are donor costs, while costs to vendors and customers are at the provider and patient costs, respectively.

Table 11. Commodity costs across the supply chain

Commodity	Cost at Level of Distribution (USD)						Source / triangulated data
	Wholesale			Retail *	Ven-dor	Consu-mer	
	Cost per unit	Packaging and labor	Total				
ACT	N/A	N/A	$1.40	$0.28	$0.31	$0.52	Qualitative demographics: Vendor cost, $0.31 consumer price $0.52. MC survey, $0.53 average consumer price (range 0 - $1.10).
Other anti-malarials	N/A	N/A	$0.25	$0.26	$0.32	$0.63	$1.65. MC survey avg $0.63, range $0.22 to $1.76
Antibiotics+	$0.37	$0.08	$0.45	$0.46	$0.56	$0.93	PSI social franchising account records
Antipyretics	N/A	N/A	$0.21	$0.22	$0.26	$0.44	Purchase of 8 paracetemol tablets in the Shan state
No anti-malarial			$0.28	$0.29	$0.35	$0.58	Assumed 70% antipyretics, 30% antibiotics

* ACTs are subsidized
+ Averaged between pneumox 125 and pneumox 250 amoxicillin products.

<u>Patient and provider time and travel costs</u>

Table 12 describes annual patient and provider time and travel costs. Travel costs are detailed in table 13 (patient travel not included in this analysis, estimated to be $504,000 annually). Time costs are detailed in table 14. Provider time is counted as a time cost because the provider is a client of the program.

Table 12. Provider time and travel costs for RDT intervention (annual)

Costs for RDT intervention, 600 providers	First year recurrent costs				Comments
	No intervention	Arm 1	Arm 2	Arm 3	
Patient and provider time costs	$0	$53,366	$53,366	$64,498	Time spent administering RDT, visiting supply point
Provider travel costs	$0	$79,344	$79,344	$79,344	Monthly travel cost per provider to reach supply point estimated to be $11.02 per provider per month

Table 13. Patient and provider travel costs

Commodity	Patient Cost	Source
Patient travel time	$3.50 per patient per visit, not included in analysis	Travel cost: reference 21: cost study from Taikkyi township Myanmar, 2004.
Provider travel to the supply point	$11.02 per provider per month. Scaled to 600 providers: $79,334 annually	Estimated by PSI program staff members, assuming providers visit supply points once a month.

Table 14. Patient and provider time costs

	**Monthly income (USD)**	**RDT time cost***	**Additional costs**	**Source / triangulated data**
Provider	$138.04	$0.29	Time cost for provider visits**	In-depth interview demographics provide provider monthly salary. The range was between $22.05 and $330.76. Reference 40 estimates $200/month salary. Product promoters estimated each support visit is 30-60 minutes.
			Arms 1 and 2: $0.32 per provider per month. $192 for 600 providers.	
			Arm 3: $1.29 per provider per month. $774 for 600 providers per month.	
			$6.90 per provider = $4,140 for 600 providers	Monthly visit to supply points: assuming 1 day per visit.
Patient	$92.00	$0.19		Assuming patient makes 2/3 of what a provider makes.

*Assuming each RDT takes 20 minutes to conduct and each provider works 40 hours per week.
** Provider visit time estimated to be 45 minutes per visit.

6. One-way sensitivity analysis

Table 15. One-way sensitivity analysis for parameters that affect cost

Parameter description	Input low, high	Cost low, high	Absolute change in costs	Change in costs per 1,000 people	Range justification
Arm 1 program costs per febrile individual (base case $3.61)	2, 10	$393,041, $1,545,041	$1,152,000	**$8,000**	Estimated from half of current range to 2.5x more
Arm 2 program costs per febrile individual (base case $3.61)	2, 10	$393,469, $1,545,469	$1,152,000	**$8,000**	
Arm 3 Program Costs per febrile individual (base case $4.23)	2, 10	$412,656 , $1,564,656	$1,152,000	**$8,000**	
Probability of using RDT for Arm 2 (base case 0.02)	0, 0.65	$622,580 , $744,847	$122,267	**$849**	From below base case to mystery client levels (0.64)
Number of febrile patients seeking care per private sector provider per month, No intervention (base case 20)	1, 40	$4,850, $858,996	$854,146	**$5,932**	Based on discussion with PSI Myanmar employees
Probability of using RDT for Arm 1 (base case 0.02)	0, 0.65	$622,152 , $730,487	$108,335	**$752**	From below base case to mystery client levels (0.64)
Probability of using RDT for Arm 3 (base case 0.08)	0.02, 0.65	$725,217 , $821,005	$95,788	**$665**	
Cost of no antimalarial (same across all arms), No intervention (base case 0.58)	0.3, 1	$60,212, $152,172	$91,960	**$639**	From cost of 5 paracetemol tables ($0.30) to above antibiotic ($0.93)
Probability of no antimalarial administration for clinical diagnosis, arm 2** (base case 0.81)	0.5, 0.93	$673,152 , $608,222	$64,929	**$451**	From below mystery client survey levels (0.63) to household survey levels (0.99, but fixed variable** makes 0.93 maximum possible value for analysis)
Probability of no antimalarial administration for clinical diagnosis, arm 1** (base case 0.81)	0.5, 0.93	$672,295 , $607,366	$64,929	**$451**	
Probability of no antimalarial administration for clinical diagnosis, arm 3** (base case 0.74)	0.5, 0.93	$768,360 , $707,406	$60,954	**$423**	
Probability of ACT administration for clinical diagnosis, no intervention** (base case 0.19)	0.05, 0.4	$614,916 , $667,765	$52,849	**$367**	Wide estimate including household survey (0.13) and mystery client (0.25) with small samples
Cost of ACT (same across all arms), arm 3 (base case $1.65)	0.5, 2.5	$704,153 , $756,651	$52,498	**$365**	Commodity base cost ($0.53) to cost without subsidy ($2.50)
Probability of using RDT for No Intervention (base case 0.02)	0.02, 0.11	$96,996 , $123,553	$26,557	**$184**	Household survey (0.02) to mystery client (0.11)

Cost of other antimalarial (same across all arms), arm 1 (base case $0.55)	0.18, 1.65	$730,476 , $745,824	$15,348	**$107**	Minimum cost to mystery clients ($0.22) to ACT societal cost ($1.65)
Cost of RDT, arm 3 (base case $0.68)	0.36, 1.2	$730,653, $740,330	$9,677	**$67**	Donor cost ($0.36) to societal cost without subsidy ($1.16)
RDT time cost (same across all arms), arm 3 (base case $0.29)	0.18, 0.75	$730,883, $737,450	$6,566	**$46**	Estimate
Probability of ACT administration for pan -, falc – test result, arm 3** (base case 0.022)	0.02, 0.4	$734,318, $738,424	$4,107	**$29**	Mystery client (0.02) to no intervention SPH survey (0.4)
Cost of RDT no intervention, arm 3 (base case $1.16)	0.36, 1.2	$94,692, $97,111	$2,419	**$17**	Donor cost ($0.36) to societal cost without subsidy ($1.16)
Cost of RDT arm 1 (base case $0.68)	0.36, 1.2	$624,564, $626,983	$2,419	**$17**	
Cost of RDT arm 2 (base case $0.80)	0.36, 1.2	$625,075, $627,494	$2,419	**$17**	
Probability of ACT administration for pan -, falc – test result, arm 2** (base case 0.083)	0.02, 0.4	$626,172, $627,199	$1,027	**$7**	Mystery client (0.02) to no intervention SPH survey (0.4)
Probability of ACT administration for pan -, falc – test result, arm 1** (base case 0.057)	0.02, 0.4	$625,386, $626,412	$1,027	**$7**	
Specificity of P. falciparum RDT arm 3 (base case 0.97)	0.92, 0.98	$734,834, $734,240	$593	**$4**	Estimate within referenced ranges for other brands
Probability of ACT administration for pan +, falc – test result, arm 3** (base case 0.1)	0.1, 0.5	$734,339, $734,551	$211	**$1**	Household survey (0.1) to SPH survey for no intervention (0.6)
Probability of other antimalarial administration for pan +, falc – test result, arm 3** (base case 0.45)	0.25, 0.45	$734,448, $734,312	$136	**$1**	No intervention (0.25) to present value (0.45)
Percentage of febrile illnesses that are malaria, no intervention (base case 8%)	0.03, 0.2	$96,958, $97,087	$130	**$1**	Wide range estimate
Probability of ACT administration for pan +, falc + test result, arm 3** (base case 0.87)	0.75, 0.9	$734,267, $734,358	$91	**$1**	No intervention (0.75) to above present value
Probability of ACT administration for pan -, falc + test result, arm 3** (base case 0.87)	0.75, 0.9	$734,293, $734,351	$58	**$0**	
Probability of ACT administration for pan +, falc – test result, arm 2** (base case 0.1)	0.1, 0.5	$626,342, $626,395	$53	**$0**	Present value to result in no intervention (0.5)
Probability of ACT administration, pan +, falc – test result, arm 1** (base case 0.1)	0.1, 0.5	$625,486, $625,538	$53	**$0**	
Specificity of pan plasmodium RDT (base case 0.98)	0.95, 1	$734,362, $734,324	$37	**$0**	Range of reported specificity for the two commodities used for intervention

Probability of other antimalarial administration for pan +, falc – test result, arm 1*** (base case 0.45)	0.25, 0.5	$625,513, $625,479	$34	**$0**	No intervention value (0.25) to above present value
Probability of other antimalarial administration arm 2*** (base case 0.45)	0.25, 0.5	$626,369, $626,335	$34	**$0**	
Sensitivity of P. falciparum RDT arm 3 (base case 1)	0.95, 1	$734,314, $734,339	$25	**$0**	Estimate within range of reported sensitivity for other brands
Percentage of falciparum vs vivax malaria arm 3 (base case 65%)	0.3, 0.7	$96,974, $96,999	$25	**$0**	Wide estimate to account for changing epidemiology given targeting of falciparum malaria in Myanmar
Probability of other antimalarial administration for pan -, falc – test result, arm 3* (base case 0.089)	0.02, 0.1	$734,360, $734,336	$24	**$0**	No intervention (0.02) to above present value
Probability of ACT administration for pan +, falc + test result, arm 1** (base case 0.78)	0.75, 0.9	$625,481, $625,504	$23	**$0**	No intervention (0.75) to above arm 3 value (0.87)
Probability of ACT administration for pan +, falc + test result, arm 2** (base case 0.84)	0.75 0.9	$626,329, $626,351	$23	**$0**	
Probability of ACT administration for pan -, falc + test result, arm 1** (base case 0.78)	0.75, 0.9	$625,483, $625,497	$14	**$0**	
Probability of ACT administration for pan -, falc + test result, arm 2** (base case 0.84)	0.75, 0.9	$626,334, $626,348	$14	**$0**	No intervention (0.02) to above arm 3 value (0.09)
Probability of other antimalarial administration for pan -, falc – test result, arm 2* (base case 0.056)	0.02, 0.1	$626,345, $626,339	$6	**$0**	No intervention (0.02) to above present value
Probability of other antimalarial administration for pan -, falc – test result, arm 1* (base case 0.03)	0.02, 0.1	$625,486, $625,480	$6	**$0**	
Sensitivity of Pan Plasmodium RDT (base case 0.92)	0.85, 1	$734,338, $734,341	$3	**$0**	Estimate within range of reported sensitivity for other brands
Probability of other antimalarial administration fo pan +, falc + test result, arm 3* (base case 0.05)	0.05, 0.13	$734,339, $734,338	$1	**$0**	From base case (0.05) to highest possible value with ACT kept constant*
Probability of other antimalarial administration for pan -, falc + test result, arm 3* (base case 0.05)	0.05, 0.13	$734,339, $734,338	$1	**$0**	
Probability of other antimalarial administration for pan +, falc + test result, arm 1* (base case 0.05)	0.05, 0.2	$625,486, $625,485	$1	**$0**	

* Holding ACT constant
** Holding other antimalarial constant
*** Holding no antimalarial constant
Note: intervention arm shown is the one that affects costs the most when cost changes are similar across arms.

Table 16. One-way sensitivity analysis on parameters that affect health outcomes

Parameter description	Input low, high	DALY low, high	Abs. change in DALYS	Change in DALYs per 1000 people	Range justification
Probability of death for nonmalarial fever given no antimalarial, no intervention (base case 0.0016)	0.001, 0.05	8,626, 133,491	124,865	**867**	From below base case to high virulence (more than untreated P. falciparum, 0.03)
Probability of death for nonmalarial fever given ACT (base case 0.002)	0.001, 0.05	8,659, 33,101	24,442	**170**	
Number of febrile patients seeking care per private sector per month (base case 20)	1, 40	508, 20,310	19,802	**138**	Based on discussion with PSI Myanmar employees
Percentage of febrile illnesses that are malaria (base case 8%)	0.03, 0.2	6,931, 17,893	10,962	**76**	Wide range estimate
Probability of death for nonmalarial fever given other antimalarial (base case 0.002)	0.001, 0.05	8,957, 18,803	9,846	**68**	From below base case to high virulence (more than untreated P. falciparum, 0.03)
Discount rate, arm 3 (base case 3%)	0, 0.05	16,408, 7,801	8,607	**60**	From none to highest discount rate typically published
Probability of death for falciparum malaria given no antimalarial, no intervention (base case 0.03)	0.005, 0.04	6,286, 11,702	5,416	**38**	Wide range estimate to capture low to high virulence
Life expectancy in Myanmar, no intervention (base case 62)	50, 80	7,846, 12,392	4,546	**32**	Wide range estimate to capture potential future development
Age of malaria death, no intervention (base case 30)	5, 45	11,342, 7,866	3,476	**24**	Wide range estimate
Probability of using RDT for Arm 2 (base case 0.02)	0, 0.65	9,777, 7,227	2,549	**18**	From below base case to mystery client levels (0.64)
Probability of using RDT for Arm 1 (base case 0.02)	0, 0.65	9,777, 7,378	2,399	**17**	
Probability of using RDT for Arm 3 (base case 0.08)	0.02, 0.65	9,386, 6,992	2,394	**17**	
Percentage of *P. falciparum* vs *P. vivax* malaria, no intervention (base case 65%)	0.3, 0.7	8,473, 10,395	1,922	**13**	Wide estimate to account for changing epidemiology given targeting of falciparum malaria in Myanmar
Probability of no antimalarial administration with clinical diagnosis, arm 1** (base case 0.81)	0.5, 0.93	8,337, 10,232	1,895	**13**	From below mystery client survey levels (0.63) to household survey levels (0.99, but fixed variable** makes 0.93 maximum possible
Probability of no antimalarial administration with clinical diagnosis, arm 2** (base case 0.81)	0.5, 0.93	8,332, 10,227	1,895	**13**	
Probability of no antimalarial administration with clinical	0.5, 0.93	8,165, 9,944	1,779	**12**	

diagnosis, arm 3** (base case 0.74)					value for analysis)
Probability of death for falciparum malaria given ACT, arm 3 (base case 0.0001)	0.0001, 0.04	9,158, 10,825	1,667	**12**	Wide range to account for potential worst case scenarios of complete drug resistance
Probability of death for vivax malaria given no antimalarial, no intervention (base case 0.01)	0.001, 0.02	9,404, 10,990	1,586	**11**	Wide range estimate to capture fatality probabilities for P. vivax malaria
Probability of ACT administration for clinical diagnosis, no intervention*** (base case 0.19)	0.05, 0.4	10,011, 8,469	1,542	**11**	Wide estimate including household survey (0.13) and mystery client (0.25) with small samples
Probability of death for falciparum malaria given other antimalarial, arm 2 (base case 0.007)	0.005, 0.04	9,675, 10,091	416	**3**	Wide range to account for potential drug resistance
Probability of using RDT for No Intervention (base case 0.02)	0.02, 0.11	10,155, 9,823	332	**2**	Household survey (0.02) to mystery client (0.11)
Weeks of febrile illness for nonimmediate recovery, arm 3 (base case 1)	0.5, 2	9,094, 9,287	193	**1**	Based on conversation with PSI Myanmar employees
DALY weight of nonmalarial fever, arm 3 (base case 0.18)	0.05, 0.4	9,087, 9,277	190	**1**	Wide range estimate to capture a range of virulence for nonmalarial fevers
Probability of death for vivax malaria given ACT, arm 3 (base case 0.0001)	0.0001, 0.01	9,158, 9,326	168	**1**	Wide estimate to account for potential drug resistance
Probability of death for vivax malaria given other antimalarial, arm 3 (base case 0.0001)	0.0001, 0.01	9,150, 9,238	89	**1**	
Probability of ACT administration, Pan + falc + test result, arm 3** (base case 0.87)	0.75, 0.9	9,204, 9,147	57	**0**	No intervention (0.25) to above present value
Probability of ACT administration, Pan – falc – test result, arm 3** (base case 0.022)	0.02, 0.4	9,158, 9,201	43	**0**	Mystery client (0.02) to no intervention SPH survey (0.4)
Probability of ACT administration, Pan + falc – test result, arm 3** (base case 0.1)	0.1, 0.5	9,158, 9,133	25	**0**	Household survey (0.1) to near SPH survey for no intervention (0.6)
Probability of other antimalarial administration, Pan + falc + test result, arm 3* (base case 0.05)	0.05, 0.13	9,158, 9,135	23	**0**	From base case (0.05) to highest possible value with ACT kept constant*
Probability of ACT administration Pan + falc + test result, arm 1** (base case 0.78)	0.75, 0.9	9,706, 9,692	14	**0**	No intervention (0.75) to above arm 3 value (0.87)

Probability of ACT administration Pan + falc + test result, arm 2** (base case)	0.75, 0.9	9,707, 9,693	14	**0**	
Probability of other antimalarial administration Pan + falc + test result, arm 1* (base case 0.05)	0.05, 0.2	9,703, 9,692	11	**0**	From base case (0.05) to highest possible value with ACT kept constant*
Probability of ACT administration Pan – falc – test result, arm 2 (base case 0.083)**	0.02, 0.4	9,697, 9,707	11	**0**	Mystery client (0.02) to no intervention SPH survey (0.4)
Probability of ACT administration Pan – falc – test result, arm 1** (base case)	0.02, 0.4	9,702, 9,713	11	**0**	
Sensitivity of P. falciparum RDT, arm 3 (base case 1)	0.95, 1	9,168, 9,158	10	**0**	Estimate within range of reported sensitivity for other brands
Probability of other antimalarial administration, Pan – falc – test result, arm 3* (base case 0.089)	0.02, 0.1	9,150, 9,159	9	**0**	No intervention (0.02) to above present value
Probability of other antimalarial administration Pan + falc + test result, arm 2* (base case 0.05)	0.05, 0.15	9,698, 9,691	7	**0**	From base case (0.05) to highest possible value while keeping ACTconstant*
Probability of ACT administration Pan + falc – test result, arm 1** (base case 0.1)	0.1, 0.5	9,703, 9,697	6	**0**	Present value to result in no intervention (0.5)
Probability of ACT administration, Pan + falc – test result, arm 2** (base case 0.1)	0.1, 0.5	9,698, 9,692	6	**0**	
Probability of ACT administration Pan – falc + test result, arm 3** (base case 0.87)	0.75, 0.9	9,162, 9,157	4	**0**	No intervention (0.75) to above present value
Sensitivity of Pan Plasmodium RDT, arm 3 (base case 0.92)	0.85, 1	9,160, 9,156	4	**0**	Estimate within range of reported sensitivity for other brands
Specificity of P. falciparum RDT, arm 3 (base case 0.97)	0.92, 0.98	9,161, 9,157	4	**0**	Estimate within referenced ranges for other brands
Specificity of pan plasmodium RDT, arm 3 (base case 0.98)	0.95, 1	9,160, 9,157	3	**0**	Range of reported specificity for the two commodities used for interventino

* Holding ACT constant
** Holding other antimalarial constant
*** Holding no antimalarial constant
Note: intervention arm shown is the one that affects DALYs the most when DALY changes are similar across arms.

7. Results beyond first year of intervention

Results presented in the main text of the paper focus on the first year of the intervention. For recurrent years, costs are lower as staff will not need new training, community outreach will not be performed, and less providers will have to be recruited. Costs for the first year and subsequent years is shown in the tables below.

Table 17. Annual commodities, programmatic expenses, time and travel cost

**Scenario (societal)**		**Total cost**	**Drug and RDT costs (scaled to uptake)**		**Program staff and non-commodity costs**	**Patient and provider time costs**	**Provider travel costs**[++]
			**Total**	**(RDT Donor only)**			
No intervention		$96,996	$95,614	**$0**	**$0**	$1,382	$0
Arm 1: simple subsidy	First year cost	$625,486	$103,658	**$1,037**	**$387,735**	$54,748	$79,344
	Recurrent annual cost	$566,152	$103,658	**$1,037**	**$328,401**	$54,748	$79,344
Arm 2: subsidy with financial incentives	First year cost	$626,342	$104,087	**$1,382**	**$388,163**	$54,748	$79,344
	Recurrent annual cost	$567,008	$104,087	**$1,382**	**$328,829**	$54,748	$79,344
Arm 3: subsidy with IEC	First year cost	$734,339	$119,127	**$4,147**	**$476,973**	$58,896	$79,344
	Recurrent annual cost	$675,005	$119,127	**$4,147**	**$417,639**	$58,896	$79,344

*Includes time spent conducting RDT, and provider time for monthly supply point visit based on wages, as providers were not compensated by the programme.

**Patient travel costs were excluded and the same across each arm, estimated to be $504,000 per arm.

Table 18. Cost-effectiveness ratios from a societal perspective

Subsidy scheme	Total costs	Added costs *vs* prior strategy	Total DALYs incurred	DALYs averted *vs* prior strategy	Incremental cost per DALY averted *vs* prior strategy	Cost per DALY averted *vs* no intervention
**Year 1**						
No intervention	$96,996	--	10,155	--	--	--
Arm 1: Simple subsidy	$625,486	$528,490	9,703	452	$1,169	$1,169
Arm 2: Subsidy with financial incentive	$626,342	$857	9,698	5	$185	$1,159
Arm 3: Subsidy with IEC	$734,339	$107,997	9,158	540	$200	$639
**Year 2 and after**						
No intervention	$96,996	--	10,155	--	--	
Arm 1: Simple subsidy	$566,152	$469,156	9,703	452	$1,038	$1,038
Arm 2: Subsidy with financial incentive	$567,008	$857	9,698	5	$185	$1,029
Arm 3: Subsidy with IEC	$675,005	$107,997	9,158	540	$200	$580

*PSI operational costs. Exchange rate used: 907 Kyat/USD, 1 May' 2013.

8. Supporting information references:

1. Give Well: Containment of artemisinin resistance in eastern Myanmar (PSI Myanmar project) http://www.givewell.org/PSI-Myanmar-Artemisinin-Resistance-Containment (accessed July 13, 2013).
2. Foundation of Innovative New Diagnostics, http://www.finddiagnostics.org/resource-centre/reports_brochures/malaria-diagnostic-test-report.html (Accessed February 7, 2013).
3. Zhou M., Liu Q., Wongsrichanalai C., Suwonkerd W., Panart K., Prajakwong S., Pensiri A., Kimura M., Matsuoaka H., Ferreira M.U., Isomura S., Kawamoto F. High prevalence of *Plasmodium malariae* and *Plasmodium ovale* in malaria patients along the Thai-Myanmar border, as revealed by acridine orange staining and PCR-based diagnoses. *Tropical Medicine and International Health* 1998, **3**: 304-312.
4. Takeuchi R., Lawpoolsri S., Imwong W., Kobayashi J., Kaewkungwal J., Pukrittayakamee S., Puangsa-art S., Thanyavanich N., Maneeboonyang W., Day N. P. J., Singhasivanon P. Directly-observed therapy (DOT) for the radical 13-day primaquine treatment of *Plasmodium vivax* malaria at the Thai-Myanmar border. *Malaria Journal* 2010, **9**:308.
5. Lwin, M. M., Sudhinaraset, M., San, A. K., Aung, T. Improving malaria knowledge and practices in rural Myanmar through a village health worker intervention: a cross-sectional study. *Malaria Journal* 2014, **13**:5.
6. Oanda currency converter. http://www.oanda.com/currency/converter/ (accessed July 13, 2013).
7. WHO SEARO 2006. http://www.who.int/malaria/publications/country-profiles/2008/mal2008-myanmar-en.pdf (Accessed December 8, 2012).
8. Myint, H.Y., Adjuik, M., Olliaro, P., Pukrittayakamee, S., Looareesuwan, S., Hien, T.T., Farrar, J., Nosten, F., Day, N.P., White, N.J. In vivo assessment of drug efficacy against Plasmodium falciparum malaria: duration of follow-up. Antimicrobial Agents and Chemotherapy 2004, 48:4271-80.
9. Ohnmar, Tun-Min, May-Aye-Than, San-Shwe, Wai-Wai-Mying, Chongsuvivatwong, V. Access to a blood test and antimalarials after introducing rapid diagnostic tests in rural Myanmar: initial experience in a malaria endemic area. *International Health* 2010, **2**:275-281.
10. Richards A. K., Banek K., Mullany L. C., Lee C. I., Smith L., Eh Kalu Shwe Oo, Lee, T. J. Cross-border malaria control for internally displaced persons: observational results from a pilot programme in eastern Burma/Myanmar. *Tropical Medicine and International Health* 2009, **14**:512-521.
11. Price, R.N., Douglas, N.M., Anstey, N.M. New developments in Plasmodium vivax malaria: severe disease and the rise of chloroquine resistance. Current Opinion in Infectious Disease 2009, 5:430-5.
12. Tijtra E., Anstey N.M., Sugiarto P., Warikar N., Kenangalem E., Karyana M., Lampah D. A., Price, R. N. Multidrug-resistant *Plasmodium vivax* associated with severe and fatal malaria: a prospective study in Papua, Indonesia. *Plos Medicine* 2008, **5**: e128.
13. Ohnmar, Tun-Min, San-Shwe, Than-Win, Chongsuvivatwong, V. Effects of malaria volunteer training on coverage and timeliness of diagnosis: a cluster randomized controlled trial in Myanmar. *Malaria Journal* 2012, **11**:309.
14. WHO Global Burden of Disease Death Estimates 2008 http://www.who.int/healthinfo/global_burden_disease/2004_report_update/en/index.html (Accessed February 10, 2013).
15. Mayxay M., Castonguay-Vanier J., Chansamouth V., Dubot-Pérès A., Paris D. H., Phetsouvanh, Tangkhabuanbutra J., Douangdala P., Inthalath S., Souvannasing P., Slesak G., Tongyoo N., Chanthongthip A., Panyanouvong P., Sibounheuang B., Phommasone K., Dohnt M., Phonekeo D., Hongvantong B., Xayadeth S., Ketmayoon P., Blacksell S. D., Moore C. E., Craig S. B., Burns M., Sonnenburg F., Corwin A., Lamballerie X., González I. J., Christophel E. M., Cawthorne A., Bell D., Newton P. N. Causes of non-malarial fever in Laos: a prospective study. *The Lancet* 2013, **1**:e46-54.

16. Ejov M. N., TUn T., Aung S., Lwi S., Sein K. Hospital-based study of severe malaria and associated deaths in Myanmar. *Bulletin of the World Health Organization* 1999, **77**: 4.
17. Lopez A.D., Mathers C.D.. Measuring the global burden of disease and epidemiological transition: 2002-2030. *Annals of Tropical Medicine and Parasitology*, 2006, **100**:481-499.
18. Murray, C.J.L., Lopez A. D., Black, R., Mathers, C. D., Shibuya, K., Ezzati, M., Salomon, J. A., Michaud, C. M., Walker, N., Vos, T. Global burden of disease 2005: call for collaborators. *The Lancet* 2007, **370**:109-110.
19. The World Bank Myanmar. http://data.worldbank.org/country/myanmar (Accessed July 19, 2013).
20. Salomon, J. A., Wang, H., Freeman, M. K., Vos, T., Flaxman, A. D., Lopez, A. D., & Murray, C. J. Healthy life expectancy for 187 countries, 1990–2010: a systematic analysis for the Global Burden Disease Study. *The Lancet*, 2013, **380:**2144-2162.
21. Wang, H., Dwyer-Lindgren, L., Lofgren, K. T., Rajaratnam, J. K., Marcus, J. R., Levin-Rector, A., Murray, C. J. et al. Age-specific and sex-specific mortality in 187 countries, 1970–2010: a systematic analysis for the Global Burden of Disease Study 2010. *The Lancet* 2013, **380**:2071-2094.
22. Cho-Min-Naing, Gatton M. L. Costs to the patient for seeking malaria care in Myanmar. *Acta Tropica* 2004, **92**:173-177.
